# Acute Effects of Caffeine on Heart Rate Variability, Blood Pressure and Tidal Volume in Paraplegic and Tetraplegic Compared to Able-Bodied Individuals: A Randomized, Blinded Trial

**DOI:** 10.1371/journal.pone.0165034

**Published:** 2016-10-24

**Authors:** Joelle Leonie Flueck, Fabienne Schaufelberger, Martina Lienert, Daniela Schäfer Olstad, Matthias Wilhelm, Claudio Perret

**Affiliations:** 1 Institute of Sports Medicine, Swiss Paraplegic Centre, Nottwil, Switzerland; 2 Institute of Human Movement Sciences and Sport, ETH Zurich, Switzerland; 3 Division of Cardiovascular Prevention, Rehabilitation and Sports Medicine, University Clinic for Cardiology, Inselspital, University Hospital and University of Bern, Bern, Switzerland; Vanderbilt University, UNITED STATES

## Abstract

Caffeine increases sympathetic nerve activity in healthy individuals. Such modulation of nervous system activity can be tracked by assessing the heart rate variability. This study aimed to investigate the influence of caffeine on time- and frequency-domain heart rate variability parameters, blood pressure and tidal volume in paraplegic and tetraplegic compared to able-bodied participants. Heart rate variability was measured in supine and sitting position pre and post ingestion of either placebo or 6 mg caffeine in 12 able-bodied, 9 paraplegic and 7 tetraplegic participants in a placebo-controlled, randomized and double-blind study design. Metronomic breathing was applied (0.25 Hz) and tidal volume was recorded during heart rate variability assessment. Blood pressure, plasma caffeine and epinephrine concentrations were analyzed pre and post ingestion. Most parameters of heart rate variability did not significantly change post caffeine ingestion compared to placebo. Tidal volume significantly increased post caffeine ingestion in able-bodied (p = 0.021) and paraplegic (p = 0.036) but not in tetraplegic participants (p = 0.34). Systolic and diastolic blood pressure increased significantly post caffeine in able-bodied (systolic: p = 0.003; diastolic: p = 0.021) and tetraplegic (systolic: p = 0.043; diastolic: p = 0.042) but not in paraplegic participants (systolic: p = 0.09; diastolic: p = 0.33). Plasma caffeine concentrations were significantly increased post caffeine ingestion in all three groups of participants (p<0.05). Plasma epinephrine concentrations increased significantly in able-bodied (p = 0.002) and paraplegic (p = 0.032) but not in tetraplegic participants (p = 0.63). The influence of caffeine on the autonomic nervous system seems to depend on the level of lesion and the extent of the impairment. Therefore, tetraplegic participants may be less influenced by caffeine ingestion.

**Trial Registration:**

ClinicalTrials.gov NCT02083328

## Introduction

It is already well-known, that a spinal cord injury implies some crucial physiological adaptations due to the injury. Such consequences include not only bone mass, active muscle mass or the inability to use the limbs, but also gastrointestinal transition time, bladder, bowel and sexual functions as well as cardiovascular capacity and the activation of the autonomous nervous system [1–6]. As the sympathetic nerves leave the spine between Th1 and L2, loss of function highly depends on the level and severity of the lesion. Whereas in paraplegic patients sympathetic nerve activity is almost fully preserved, tetraplegic patients mostly suffer from the inability to activate sympathetic nervous system [7].

Heart rate variability (HRV) is a non-invasive measure of markers of cardiac autonomic modulation. With power spectral analysis, R-peak to R-peak intervals (RRI) of consecutive heart beats can be divided into low frequency (LF) and the high frequency (HF) power. It is generally accepted that HF is a marker of cardiac parasympathetic activity [8] and that LF fluctuations of HRV at rest are not related to muscle sympathetic nerve activity [9]. However, when measured in an orthostatic challenge, it has been shown that LF/HF power ratio and muscle sympathetic nerve activation change in parallel [10, 11], suggesting that this HRV ratio may reflect enhanced adrenergic activity as response to provoked stress.

It was previously shown that HRV measurements are reproducible in able-bodied as well as in spinal cord injured participants [12]. Bunten, Warner [13] found that HRV is altered following a spinal cord injury mainly due to a loss of sympathetic nervous tone corresponding to a loss of LF. Some other studies investigated HRV during exercise [14–16], in head-up tilt [17] or during rest [18–21] in paraplegic and tetraplegic individuals. Inoue, Ogata [22] found no LF in 9 out of 15 tetraplegic participants which confirms that LF implies not only a sympathetic component but also parasympathetic part [8]. As caffeine acts as a stimulant, it appeared to be a good tool to further investigate its influence on the autonomous nervous system in paraplegic and tetraplegic individuals [23, 24]. Furthermore, caffeine was shown to have some ergogenic effects on exercise performance in various sports disciplines [25–27]. Therefore, its effects on the autonomic nervous system need to be investigated before an application in athletes with a spinal cord injury as well. Investigating the effects of caffeine on the autonomic nervous system in individuals with a spinal cord injury will therefore provide more information about the mechanism of action of this stimulant. In addition, it might help to evaluate whether this stimulant in such a high dose is still safe in these subjects. Therefore, our aim was to investigate, whether caffeine alters HRV, blood pressure and plasma epinephrine and norepinephrine concentrations compared to placebo in paraplegic and tetraplegic patients compared to able-bodied participants. Additionally, we aimed to assess the differences in baseline HRV between high lesioned tetraplegic, low lesioned paraplegic and able-bodied participants. As HRV was known to be influenced by respiration [28] and caffeine induces bronchodilation [29], tidal volume was recorded during metronomic breathing as it might confound HRV results.

## Materials and Methods

### Participants

A double-blind, placebo-controlled and randomized study design was chosen (S1 File). Participants were included if they were healthy, non-smoking men between 18 and 60 years old. They had to be physically active for a minimum of three times 45 minutes per week. All participants with a spinal cord injury were motor and sensory complete lesioned. Participants with a paraplegia showed a lesion level below Th10 and participants with a tetraplegia a lesion level between C5 and C7. Drugs affecting the cardiovascular function were not allowed whereas the intake of any other drugs was kept constant throughout the trials. Participants suffering from diabetes were excluded from study participation. No changes were made to the study protocol after study commencement.

During the testing phase, participants followed their habitual training patterns and did not increase or decrease training volume. Light training sessions were performed the last two days prior the trial. Participants didn`t drink any alcohol 24 hours before the test session. They abstained from caffeine on the test day. The diet during the study phase was self-selected and ad libitum. Participants were asked to eat breakfast exactly 2 hours before the start of the measurements and to replicate the meal on the second trial. A nutrition and exercise protocol was filled out together with the participant before the start of the test session to check if the participants followed these instructions. Participants were asked to sleep at least seven hours the two nights before the measurements. They were excluded from data analysis if they violated any of these conditions. All tests were performed in our sports medicine laboratory where temperature (22°C) and humidity (40%) were kept constant. The two tests were performed at least 2 days and at most 2 weeks apart from each other at the same time of the day.

The study was approved by the local ethical committee (Ethikkommission Nordwest- und Zentralschweiz, EKNZ, Basel, Switzerland) and written informed consent was obtained from the participants prior starting the study. All procedures (S2 File) were in accordance with the ethical standards of the institutional and national research committee and with the 1964 Helsinki Declaration.

### Experimental design

Each participant visited the laboratory on three different occasions. On the first visit written informed consent was obtained. A questionnaire for medical history was used to check if the participants fulfilled inclusion criteria and therefore, to mitigate the cardiopulmonary risk. They were asked for their regular caffeine consumption (i.e. how often and how many cups of coffee, black tee, energy drinks per week?) and their sports activity. Caffeine intake was then quantified taking into account the frequency, the amount and the product consumed by the participant per week and then divided by seven to get the average daily caffeine consumption. A first measurement for HRV was performed to become accustomed to the experimental protocol and to check fixing of the spinal cord injured individuals. In addition, a ramp test to exhaustion at an arm crank ergometer (Ergoline GmbH, Bitz, Germany) starting at 20 W with an increment of 1 W every 6 s was used to determine VO_2peak_ (Oxycon Pro, CareFusion Germany, Hoechberg, Germany).

On the second and third visit, participants were placed onto a couch in supine position in a dark and quiet room and rested for 10min. Afterwards, HRV assessment started in supine followed by sitting position. Subsequently, a blood sample was taken from the antecubital vein. Thereafter, the participant had to ingest a gelatin capsule either containing placebo or caffeine. A break of 40 min provided enough time for absorption [30]. The ingestion of any food or performing physical activity were prohibited during this period. After the 40 min break, a second HRV assessment was performed with a 10 min resting period in supine position beforehand. Subsequently, a second blood sample was collected to analyze plasma caffeine, epinephrine and norepinephrine concentrations.

Main outcome parameters were HRV parameters before and after caffeine or placebo ingestion under resting conditions. Furthermore, we were interested in plasma epinephrine, norepinephrine and caffeine concentrations pre and post supplement ingestion. Secondary outcome parameters were tidal volume during HRV measurements as well as systolic and diastolic blood pressure.

### Heart rate variability

A HRV assessment consisted out of 6 min measurement in supine position and another 6 min in sitting position. Paced breathing (15 breaths per min, 0.25 Hz) was applied provided through and audio recording for standardization purposes. The sitting position was achieved passively by increasing the backrest up to 60°. All tetraplegic participants were able to stabilize their upper body by themselves while sitting.

The RRI were recorded using a heart rate monitor (RS800CX, Polar Electro Oy, Kempele, Finland) with a sample frequency of 1000 Hz. Data was transferred to a software (Polar ProTrainer, Polar Electro Oy, Kempele, Finland) where the information was stored as a text-file for further analysis with the Kubios software (Kubios, Department of Applied Physics, University of Eastern Finland, Kuopio, Finland). Any signals interfering with the analysis were removed using an appropriate artifact correction factor [31].

Stationary segments of at least 2 min and at most 5 min were analyzed for supine and sitting position. The length of all analyzed data segments were always the same within a participant. The segment in supine position ended at the highest point in the RRI before the decrease of the curve had started. The segment in sitting position started right after the one in supine position ended. Markers of cardiac parasympathetic activity were analyzed from the segment recorded in supine position whereas markers of sympathetic activity were analyzed from the segment recorded in sitting position. The time domain parameters, MeanRR (mean value of all RRI within a segment), SDNN (standard deviation of all normal RRI) and RMSSD (root of the mean squared differences of successive RRI), and the Fast Fourier Transformation (FFT) were used to analyze RR time series. For FFT 256 consecutive beats were analyzed. Oscillation with frequencies from 0.04 to 0.15 Hz were classified as LF and frequencies of 0.15 to 0.40 Hz as HF. The absolute power of each frequency was expressed as ms^2^ and was calculated by integrating the area under the curve. The ratio of LF to HF power (LF/HF) was calculated using the absolute power of LF and HF. HF and LF in normalized units (HFn.u. and LFn.u.) were not reported due to their redundancy with LF/HF power ratio [32].

### Blood pressure and tidal volume

Blood pressure was recorded at the left arm in the 9^th^ minute of each 10 min resting period during the first and the second HRV measurement using an automated blood pressure monitor (boso medistars, Bosch + Sohn GmbH, Jungingen, Germany).

Tidal volume was measured using a metabolic cart (Oxycon Pro, Jaeger GmbH, Höchberg, Germany). Data for tidal volume was recorded breath-by-breath during the HRV measurement and averaged over the time period of each HRV segment in supine and sitting position. Tidal volume was measured to monitor any possible influence of changing tidal volume on HRV [28].

### Blood sampling and analysis

Blood samples were taken subsequently after the HRV measurements from the antecubital vein. Blood was drawn into 7 ml Lithium-Heparin tubes (S-Monovette, Sarestedt, Sevelen, Switzerland) and centrifuged for 10 min at 4°C and 3000 rpm within a minute after blood withdrawal. Centrifuged samples were pipetted into 1.5 ml aliquots and immediately frozen in liquid nitrogen (Pangas, Dagmarsellen, Switzerland). They were stored at -80°C until analysis. All samples were sent deep frozen to an external laboratory (Institut für Klinische Chemie, Universitätsspital Zurich, Zurich, Switzerland) for analysis of caffeine concentration or to determine epinephrine and norepinephrine concentrations (Division de pharmcologie clinique, Centre Hospitalier Universitaire Vaudois, Lausanne, Switzerland). High performance liquid chromatography (HPLC) was performed to determine these concentrations.

### Caffeine and placebo administration

Randomization was applied using the data management software (SecuTrial, interActive Systems GmbH, Berlin, Germany) which randomized trials automatically. Randomization of treatment sequence with a fixed block size of 5 and stratified by group was applied. Caffeine as well as placebo were ingested in form of gelatin capsules either containing 50 or 100 mg. Placebo capsules were filled with a sugar alcohol (mannitol), which was not expected to have any further effects on performance. The caffeine capsules were filled with pure caffeine powder. Placebo and caffeine capsules were not distinguishable from each other due to equal color, size and taste.

The dosage for each participant was calculated by multiplying body mass with 6 which equates a dosage of 6 mg caffeine per kg body mass. As only 50 and 100 mg capsules were available, the dosages were then rounded up or down resulting in an actual dose varying from 5.8 to 6.2 mg/kg body mass. The number of capsules was kept identical in the placebo trial. At the end of each trial, participants were asked for their assumption concerning the type of capsules swallowed. Additionally, gastrointestinal side effects were recorded.

Neither the head of study, nor participants and staff knew the assignment of interventions during the study phase. The blinding process was done by the Clinical Trial Unit in our center where the key for trial assignment was stored.

### Statistics

A two-sided power analysis was performed. Applying a significance level of 0.05, a power of 0.8, a standard deviation of 5 Watt and an effect size of 1 resulted in an actual power of 0.84 and a total sample size of 9 participants per group. An additional over-recruitment by approximately 20% was anticipated in order to take possible drop-outs into account.

Data were tested for normal distribution using the Q-Q-plot, the Kolmogorov-Smirnov and the Shapiro-Wilk test. Results are presented as median [minimum; maximum] as data was not normally distributed. Statistical significance level was set at 0.05. To determine differences in parameters between pre and post supplement ingestion or between placebo and caffeine trials within the same group, the Wilcoxon signed-rank test was applied. The Kruskal-Wallis test was used to find any differences between the three groups whereas significant differences were then located using the Mann-Whitney-U test as a post hoc analysis. Bonferroni corrections were applied, where multiple testing with the Mann-Whitney-U test was done and the statistical significance level was then set to 0.0166. Spearman correlation was applied to find any relationship between habitual caffeine consumption and different parameter outcomes. Data are presented as the p-value and the Spearman correlation coefficient. All calculations were performed using the PSAW Statistics software (Version 18.0, SPSS Inc., Chicago, USA).

## Results

**Fig 1:**
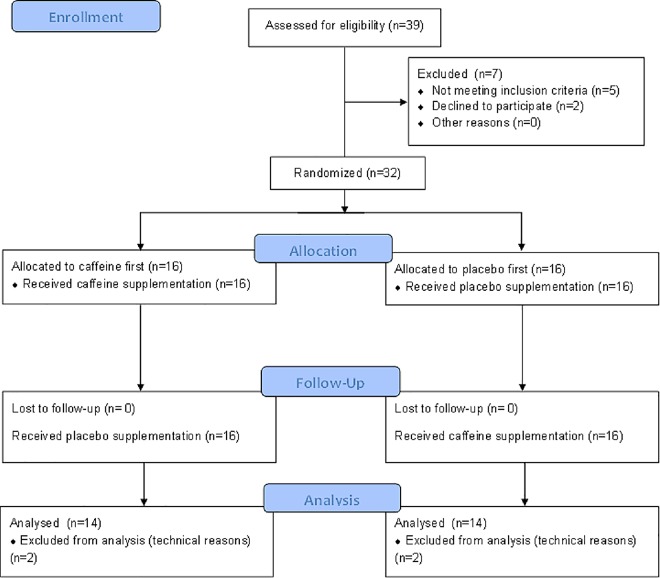
Participant recruitment flowchart.

In total 39 healthy non-smoking men were recruited to participate in the study whereas 7 must have been excluded due to not fulfilling inclusion criteria or due to participation declination. Data was analyzed finally from 28 healthy, non-smoking men (12 able-bodied, 9 paraplegic and 7 tetraplegic participants) at the end of the study (Fig 1). Data from 4 participants were excluded for analysis due to incomplete HRV measurements (technical problems). Twelve able-bodied participants were selected to form a control group (median [minimum; maximum], age: 31 y [25; 52]; height: 182 cm [172; 190]; body mass: 79 kg [67; 95]). Characteristics of paraplegic and tetraplegic individuals are shown in Table 1. They were physically active during 7 h [3; 10] (able-bodied), 8 h [3; 8] (paraplegic) and 2 h [2; 15] (tetraplegic) per week involving upper body muscles. All participants were habitual caffeine consumers with a daily caffeine intake of 250 mg [2; 600] for able-bodied, 250 mg [32; 440] for paraplegic and 200 mg [72; 420] for tetraplegic participants. Data were collected between the July 2014 and January 2015.

**Table 1:** Characteristics of spinal cord injured participants.

	Lesion level	AIS	Age[years]	Height [cm]	Weight [kg]	Time since injury [years]	VO_2_peak [ml/min/kg]
Paraplegic participants							
**1**	Th 10	A	32	183	73	16	38.4
**2**	Th 11	A	55	174	72	25	38.9
**3**	Th 11	A	59	178	80	41	19.5
**4**	Th 12	A	22	185	63	4	29.3
**5**	L 1	A	26	150	64	26	30.6
**6**	L 1	A	32	175	76	8	33.1
**7**	L 1	A	35	165	63	35	48.8
**8**	L 1	A	48	185	80	26	35.7
**9**	L 4	A	44	176	78	23	39.7
Median			43 [22; 59]	176 [150;185]	73 [63; 80]	25 [4; 41]	35.7 [19.5; 48.8]
Tetraplegic participants							
**1**	C 5	A	23	188	85	5	13.4
**2**	C 5	A	43	176	80	3	11.5
**3**	C 5	A	65	190	75	37	16.3
**4**	C 6	A	31	180	60	12	13.6
**5**	C 6	A	56	170	74	45	8.6
**6**	C 7	A	31	190	80	14	14.8
**7**	C 7	A	40	168	60	7	15.3
Median			40 [24; 65]	180 [168; 190]	75 [60; 80]	12 [3; 45]	13.6 [8.6; 16.3]

Data presented as median [minimum; maximum]; AIS = American Spinal Injury Association Impairment Scale; SCI = spinal cord injury; VO_2_peak = peak oxygen consumption, Th = thoracic; C = cervical; L = lumbar

Data for all HRV measurements pre and post ingestion of either caffeine or placebo are shown for the supine and sitting position in Tables 2 and 3. Systolic and diastolic blood pressure before and after the ingestion of any supplement are listed in Table 4. In able-bodied participants, systolic and diastolic blood pressure increased by ~9 and ~8 mmHg respectively after the ingestion of caffeine. Tetraplegic participants showed an increase of ~19 and ~27 mmHg in systolic and diastolic blood pressure. In contrast, blood pressure did not increase significantly in paraplegic participants nevertheless, the median systolic blood pressure was higher by 11 mmHg after the ingestion of caffeine.

**Table 2:** Heart rate variability (HF, LF, LF/HF and TP) pre and post ingestion of either placebo or caffeine supplementation in able-bodied, paraplegic and tetraplegic participants in supine and sitting position.

	Placebo		Caffeine		Δ p-value
	Pre	Post	Pre	Post	
***HF (supine) [ms***^***2***^***]***					
*AB*	723 [23; 3724]	1044 [25; 7154]*	689 [171; 5862]	1442 [168; 4517]	0.31
*P*	239 [20; 3376]	566 [60; 6092]*	487 [27; 1767]	659 [26; 2744]	0.07
*T*	757 [85; 6745]	784 [247; 4265]	848 [36; 2380]	666 [211; 3598]	0.87
***HF (sitting) [ms***^***2***^***]***					
*AB*	1005 [19; 6088]	1341 [16; 5426]	415 [56; 7119]	1110 [136; 7039]	0.75
*P*	162 [23; 4670]	513 [29; 6971]*	373 [34; 1882]	465 [11; 2157]	0.038^**§**^
*T*	323 [17; 824]	477 [251; 2877]	275 [19; 2553]	508 [222; 2595]	0.61
***LF (supine) [ms***^***2***^***]***					
*AB*	741 [124; 2477]	900 [99; 2581]*	375 [108; 3226]	789 [135; 3604]*	0.08
*P*	196 [53; 1865]	564 [42; 2803]	472 [69; 1272]	200 [40; 1612]	0.07
*T*	151 [14; 1180]	243 [87; 1549]	673 [10; 1018]	359 [126; 588]	0.13
***LF (sitting) [ms***^***2***^***]***					
*AB*	1064 [88; 2325]	891 [147; 3063]	747 [107; 4384]	1037 [89; 4228]	0.48
*P*	357 [56; 3651]	1025 [77; 3136]	432 [95, 1730]	334 [35; 2622]	0.09
*T*	247 [2; 1089]	363 [42; 1126]	281 [16; 967]	313 [68; 648]	0.50
***LF/HF (supine)***					
*AB*	1.08 [0.17; 5.31]	0.66 [0.18; 3.90]	0.72 [0.10; 1.01]	0.65 [0.15; 2.8]	0.31
*P*	0.90 [0.19; 13.10]	0.70 [0.29; 6.68]	0.70 [0.46; 9.65]	0.59 [0.24; 1.80]	0.95
*T*	0.25 [0.17; 0.87]	0.51 [0.18; 0.70]	0.80 [0.14; 2.75]	0.43 [0.10; 1.29]	0.31
***LF/HF (sitting)***					
*AB*	1.57 [0.21; 4.55]	1.21 [0.36; 9.06]	1.27 [0.29, 7.18]	0.63 [0.25; 3.81]	0.39
*P*	1.56 [0.60; 7.83]	1.68 [0.45; 5.06]	0.98 [0.45; 12.68]	0.57 [0.19; 6.39]*	0.37
*T*	0.44 [0.10; 1.41]	0.76 [0.07; 1.73]	0.83 [0.38; 1.95]	0.56 [0.13; 1.09]	0.61
***TP (supine) [ms***^***2***^***]***					
*AB*	1591 [180; 5938]	1901 [149; 9851]*	1159 [340; 8000]	3014 [401; 8265]*	0.53
*P*	437 [98; 5472]	1291 [106; 8391]*	1014 [189; 3120]	934 [78; 4566]	0.038^**§**^
*T*	951 [104; 7970]	1320 [442; 5894]	1346 [53; 3449]	942 [358; 4000]	0.87
***TP (sitting) [ms***^***2***^***]***					
*AB*	2158 [111; 7538]	2172 [167; 8444]	1394 [186; 11733]	2589 [264; 11739]	0.75
*P*	634 [88; 9462]	1946 [189; 10507]*	1094 [264; 3743]	1159 [95; 4934]	0.015^**§**^
*T*	663 [30; 2024]	856 [341; 4180]	611 [36; 3582]	668 [473; 3221]	0.61

*AB* = able-bodied participants; *P* = paraplegic participants, *T* = tetraplegic participants; *HF* = high frequency power; *LF* = low frequency power; *LF/HF* = ratio between LF and HF; *TP* = total power

* significant change from pre to post ingestion (p < 0.05)

^**§**^ significant difference between the changes in the placebo and the caffeine trial (p < 0.05)

Δ p-value = p-value comparing the difference between pre and post measurement of the placebo trial with the difference from the caffeine trials

**Table 3:** MeanRR, SDNN, RMSSD and heart rate during rest in sitting and supine position.

	Placebo		Caffeine		
	Pre	Post	Pre	Post	Δ p-value
***MeanRR (supine) [ms]***					
*AB*	1076 [823; 1273]	1240 [895; 1353]*	1108 [881; 1436]	1176 [1004; 1477]	0.53
*P*	922 [770; 1153]	1049 [910; 1262]*	974 [781; 1262]	1078 [857; 1449]*	0.77
*T*	1158 [908; 1326]	1247 [1036; 1415]*	1229 [829; 1355]	1347 [975; 1647]*	0.40
***MeanRR (sitting) [ms]***					
*AB*	1088 [761; 1276]	1194 [786; 1377]*	991 [803; 1401]	1130 [892; 1358]	0.94
*P*	864 [707; 1111]	906 [756; 1180]*	946 [736; 1060]	995 [758; 1286]*	0.21
*T*	1130 [789; 1307]	1142 [900; 1223]	1139 [757; 1213]	1217 [867; 1633]*	0.50
***SDNN (supine) [ms]***					
*AB*	44 [12; 82]	48 [11; 111]	34 [18; 94]	57 [18; 98]*	0.91
*P*	28 [10; 79]	36 [12; 107]	31 [13; 60]	39 [12; 69]	0.09
*T*	31 [11; 91]	38 [25; 87]	38 [8; 66]	42 [20; 65]	0.50
***SDNN (sitting) [ms]***					
*AB*	49 [14; 89]	50 [17; 95]	39 [15; 99]	53 [19; 107]	0.75
*P*	29 [13; 95]	42 [14; 95]*	33 [17; 61]	33 [12; 74]	0.09
*T*	30 [6; 42]	35 [21; 66]	26 [7; 62]	33 [22; 66]	0.61
***RMSSD (supine) [ms]***					
*AB*	50 [7; 126]	58 [9; 158]*	40 [21; 156]	71 [20; 130]	0.58
*P*	34 [7; 100]	43 [13; 136]*	35 [9; 70]	41 [9; 102]	0.11
*T*	41 [14; 130]	55 [33; 126]	52 [8; 99]	50 [27; 108]	0.74
***RMSSD (sitting) [ms]***					
*AB*	53 [10; 138]	65 [10; 147]	30 [15; 161]	54 [21; 140]*	1.0
*P*	32 [9; 100]	38 [9; 107]	32 [11; 56]	35 [7; 70]	0.44
*T*	32 [7; 51]	40 [26; 84]	29 [7; 82]	43 [23; 103]	0.73
***HR (supine) [1/min]***					
*AB*	56 [47; 73]	49 [45; 67]*	54 [42; 68]	52 [41; 61]	0.24
*P*	65 [52; 78]	57 [48; 66]*	62 [48; 77]	56 [41; 71]*	0.68
*T*	52 [45; 66]	48 [42; 58]*	49 [44; 72]	45 [36; 62]*	0.40
***HR (sitting) [1/min]***					
*AB*	56 [47; 79]	51 [44; 76]*	60 [43; 75]	54 [45; 68]	0.94
*P*	70 [54; 85]	66 [51; 79]*	64 [57; 82]	61 [47; 79]*	0.52
*T*	53 [46; 76]	53 [49; 67]	53 [50; 79]	49 [37; 69]	0.74

*AB* = able-bodied participants; *P* = paraplegic participants, *T* = tetraplegic participants; *MeanRR*, = mean of the RR interval; *SDNN* = standard deviation of all normal RR intervals; *RMSSD* = the squared root of the mean squared differences of successive RRI intervals; *HR =* heart rate

* significant change from pre to post ingestion (p < 0.05)

Δ p-value = p-value comparing the difference between pre and post measurement of the placebo trial with the difference from the caffeine trials

**Table 4:** Blood pressure pre and post ingestion of either placebo or caffeine.

	Placebo		Caffeine	
	Pre	Post	Pre	Post
*Able-bodied*				
Systolic BP	110 [92; 125]	113 [96; 124]	107 [87; 137]	116 [106; 153]*
Diastolic BP	64 [51; 82]	67 [54; 83]	64 [51; 81]	72 [66; 102]*
*Paraplegic*				
Systolic BP	108 [98; 128]	117 [98; 135]*	112 [100; 142]	123 [100; 153]
Diastolic BP	70 [53; 80]	75 [56; 83]	71 [58; 86]	73 [55; 92]
*Tetraplegic*				
Systolic BP	100 [88; 122]	101 [90; 131]	100 [94; 110]	119 [95; 127]*
Diastolic BP	59 [48; 82]	58 [45; 88]	58 [54; 71]	85 [61; 105]*

Data presented as median [minimum; maximum]; BP = blood pressure

* = significant difference compared to pre ingestion (p < 0.05)

**Fig 2:**
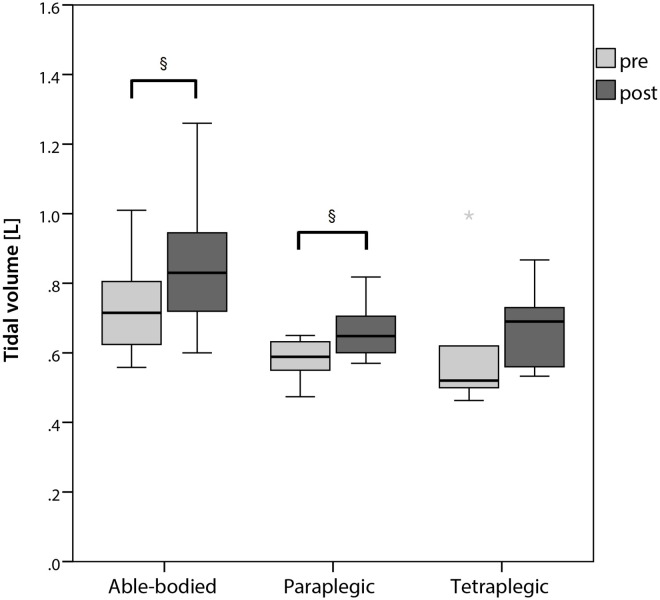
Tidal volume in supine position pre and post ingestion of caffeine. * = outlier; § = significant difference between pre and post (p < 0.05).

**Fig 3:**
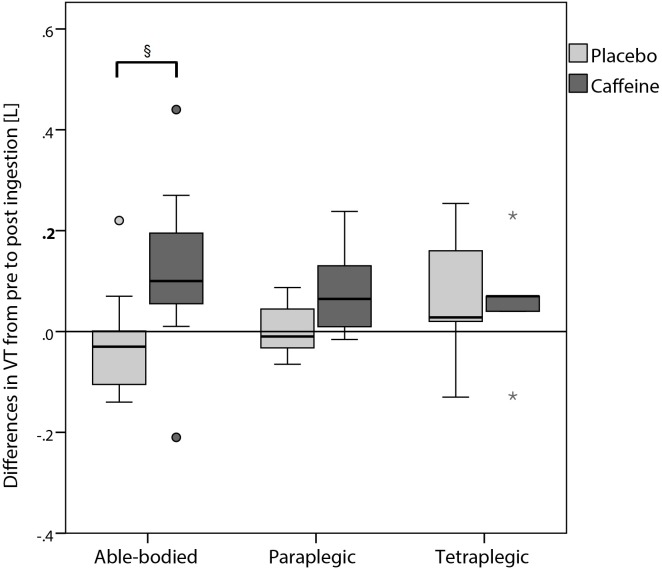
Differences in tidal volume (supine position) from pre to post ingestion between placebo and caffeine trial. ° = outlier; * = extreme outlier; § = significant difference between the change from pre to post caffeine compared to placebo ingestion (p < 0.05).

**Fig 4:**
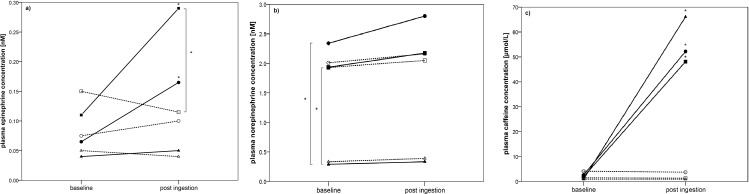
**Changes in plasma epinephrine (a), norepinephrine (b) and caffeine (c) concentrations from pre to post ingestion of the supplement illustrated for all three groups.** Dashed line represents placebo trail, bold line represents caffeine trial; ●, ○ = able-bodied, ■, □ = paraplegic and ▲, Δ = tetraplegic group.

Even though metronomic breathing was applied, tidal volume increased significantly in supine position after the ingestion of caffeine (Fig 2) compared to the measurement before the ingestion in able-bodied and paraplegic participants. Comparing the change in tidal volume in supine position from pre to post ingestion in the placebo compared to the caffeine trial, only able-bodied participants showed a significant increase (Fig 3). Fig 4 shows the change in plasma caffeine, epinephrine and norepinephrine concentrations from pre to post ingestion of either placebo or caffeine. Plasma epinephrine concentration increased significantly in able-bodied (p = 0.002) and paraplegic (p = 0.032) but not in tetraplegic participants (p = 0.63) from pre to post caffeine ingestion. Tidal volume and the difference of epinephrine concentration from pre to post caffeine ingestion were significantly correlated only in able-bodied participants (p = 0.038). Change in HF from pre to post placebo ingestion was significantly correlated with total daily caffeine consumption in able-bodied (R = 0.61; p = 0.035) and in paraplegic (R = 0.70; p = 0.036) but not in tetraplegic (R = 0.00; p = 1.00) participants. The same analysis in the caffeine trial did not show a significant correlation in any group (able-bodied: R = 0.11; p = 0.74; paraplegic: R = 0.13; p = 0.73; tetraplegic: R = 0.39; p = 0.38). The change of RMSSD in the placebo trial showed a trend to correlate with total daily caffeine consumption in able-bodied (R = 0.52; p = 0.081) and in paraplegic (R = 0.65; p = 0.058) but not in tetraplegic (p = 0.65) participants. Change in LF/HF from pre to post caffeine ingestion was not significantly correlated to the change in epinephrine concentration in the caffeine trial in any group of participants (able-bodied: p = 0.39; paraplegic: p = 0.058; tetraplegic: p = 0.67). No gastrointestinal side effects occurred.

## Discussion

Ingestion of 6 mg caffeine per kg body mass leads to an increase in blood pressure and tidal volume in able-bodied, paraplegic and tetraplegic participants. It did not change most HRV indices significantly in these participants. Even though plasma caffeine concentration was significantly increased in all three groups of participants, plasma epinephrine increased only in able-bodied participants significantly.

### Heart rate variability

To our knowledge, this study was the first one, assessing the influence of caffeine on HRV in spinal cord injured compared to able-bodied participants (Tables 2 and 3). HF significantly increased in able-bodied as well as in paraplegic participants in the placebo trial but not in the caffeine trial. LF did not change in any group at any trial. Furthermore, LF/HF ratio significantly decreased in paraplegic participants following the ingestion of caffeine. In the other two groups, a nonsignificant decrease in this ratio was found. TP increased significantly in able-bodied and paraplegic participants in the placebo trial and only in able-bodied participants in the caffeine trial. In addition, TP seemed to be lower from pre to post caffeine ingestion in paraplegic and tetraplegic participants. A decrease of the heart rate during both trials seemed to occur in all three groups, whereas it was not always a significant decrease. Overall, HRV seemed not to be substantially changed following the ingestion of caffeine in paraplegic and tetraplegic participants even though significant differences in baseline LF values between the three groups were found. Tetraplegic participants showed the smallest values, which can be explained by their impaired sympathetic function.

Hibino, Moritani [33] showed a significant increase in HF and TP in normal healthy population after consummation of 240 mg caffeine. In addition, they observed a great variability in LF/HF, whereas results couldn`t be presented. They suggested, that coffee intake induced calmness and that only the relaxing effect of caffeine was seen in the HRV. Additionally, they suppose, that an increasing tidal volume might increase HF. Therefore, it is possible, that our HF parameters in the caffeine trial were overrated due to the increase in tidal volume. No studies were found showing the impact of altered tidal volume on HRV. Monda, Viggiano [34] found an increase in HF after the ingestion of 75 mg caffeine in form of espresso but no change in LF. Similar results were found by Notarius and Floras [35] in healthy participants, whereas the ingestion of 4 mg caffeine per kg body mass increased LF, HF and TP with a decrease in LF/HF ratio. In contrast, they found no change in HRV in patients with a chronic heart failure where caffeine infusion did not alter any HRV parameter. In comparison, our results showed a trend toward a reduction of LF/HF ratio as well, even if this reduction was only significant in paraplegic participants. In contrast, LF and HF were not altered following caffeine ingestion. Two other studies [36, 37] have shown a decrease in LF/HF ratio following caffeine ingestion. Richardson, Rozkovec [36] investigated the ingestion of two times 250 mg caffeine in patients suffering from diabetes compared to a control. They showed a reduction in sympathetic and an increase in parasympathetic activity. LF/HF ratio was significantly reduced in diabetes patients following caffeine ingestion. Rauh, Burkert [37] showed a decreased LF/HF ratio following the ingestion of 200 mg caffeine. In addition, a lower heart rate was shown in all trials (even placebo), but it was not significant. These results are very similar to ours, as we have found significant reductions of heart rate in both trials in all participants. This reduction might be explained by the increase of cardiac parasympathetic activity. The increase in HF in the placebo trial in paraplegic and able-bodied participants seems to positively correlate with daily caffeine consumption. One can hypothesize, that in these trials, missing caffeine consumption did activate parasympathetic nerve activity to a greater extent than in the caffeine trial. Since Ditor, Kamath [12] showed, that HF is not reliable in spinal cord injured individuals, it is difficult to draw final conclusions concerning the effects of caffeine.

Comparing LF pre ingestion of the supplement, we detect a significant difference between our three groups. The lowest LF was found for tetraplegic participants whereas able-bodied showed the highest LF. Even for paraplegic participants, LF was significantly reduced, compared to able-bodied participants. These findings are in line with the results from Inoue, Ogata [22] where they showed only HF and no LF in 9 of 15 tetraplegic participants. The other 6 participants showed a significantly lower LF compared to 10 able-bodied controls. In paraplegic participants, both LF and HF were represented, but they were reduced compared to able-bodied controls. Furthermore, they concluded, that LF implies other physiological mechanisms than in able-bodied controls. Two other studies [13, 19] investigated HRV in spinal cord injured participants. Both studies found a lower in LF in paraplegic participants compared to able-bodied. HF seemed to be similar in all different groups of participants. Conclusively, LF seems to be present in highly lesioned tetraplegic participants showing a motor and sensory completely lesion but it seems to be significantly reduced compared to paraplegic and able-bodied participants. Thus, these results might support the theory that LF implies 25% parasympathetic and 50% sympathetic nerve activity [8].

### Blood pressure and tidal volume

Systolic and diastolic blood pressure increased in all three groups, whereas the increase was only significant in the able-bodied and tetraplegic participants (Table 4). The increase of blood pressure was the highest in tetraplegic participants with an increase of 19 mmHg in systolic and with 27 mmHg in diastolic blood pressure. Such an increase of blood pressure following the ingestion of caffeine was found in several different able-bodied studies and was reviewed by Nurminen, Niittynen [38]. Even in tetraplegic participants a significant increase in systolic and diastolic blood pressure was found 60 min and 75 min after the ingestion of caffeine [23]. Another study showed a significant increase in mean arterial pressure (MAP) in tetraplegic participants after the ingestion of 4 mg caffeine per kg body mass [39]. A case report showed that the ingestion of caffeine leads to increased blood pressure in a paraplegic participant with a lesion sub Th3 [40]. To summarize, blood pressure might be increased either due to an increase in epinephrine concentration activating the sympathetic nervous system by increasing blood pressure as well. Otherwise, caffeine might directly act on adenosine and ryanodine receptors on heart muscle tissue increasing contractility of the heart leading to an increase in stroke volume. The physiological consequence of an increased stroke volume is an increase in systolic blood pressure. From a clinical point of view, a higher blood pressure can be of interest for individuals with a spinal cord injury in two ways. First, many persons with a spinal cord injury suffer from orthostatic hypotension, which affects quality of daily living. The ingestion of caffeine might help to alleviate symptoms such as dizziness. Second, for wheelchair athletes, an increase in systolic blood pressure induces a higher cardiac output leading to a better exercise performance. thus, caffeine might be of interest as an ergogenic aid in athletes with a spinal cord injury. In fact, some studies [30, 41] were showing potential benefits of caffeine supplementation. However, further studies are needed to elucidate this issue in more detail.

Similar to our blood pressure results, tidal volume increased significantly after the ingestion of caffeine in able-bodied as well as in paraplegic participants (Figs 2 and 3). We did not find any significant increase in tidal volume in our tetraplegic participants even though it tended to be higher. Similar results were shown, when 5 mg caffeine per kg body mass was administrated [42]. A significant increase in tidal volume was shown and authors concluded, caffeine might be a respiratory stimulant. Similar findings were shown by Kraaijenga, Hutten [43], whereas intravenous caffeine administration increased tidal volume significantly in preterm infants due to increased diaphragmatic contractility. On the other hand, caffeine did not change respiratory rate. They suggested, that contractility might be increased due to augmented release of calcium in the sarcoplasmic reticulum [44]. Another possibility to increase contractility might involve the adrenal release of epinephrine concentration, which was not present in our participants. Therefore, we speculate, that if tidal volume was increased due to increased diaphragmatic contractility, the major mechanism would probably involve calcium release in the sarcoplasmic reticulum in our spinal cord injured participants. This suggestion is supported by our results, showing a significant correlation of epinephrine increase with tidal volume increase in able-bodied participants. Thus, the release of epinephrine would only play a minor role in increasing tidal volume.

### Blood parameters

Plasma epinephrine concentrations significantly increased following caffeine ingestion in able-bodied, as well as in paraplegic but not in tetraplegic participants (Fig 4). Plasma norepinephrine concentration on the other hand, did not increase due to caffeine in all three groups. Similar results were found by Mohr, Van Soeren [45] whereas one paraplegic individual was able to increase plasma epinephrine and norepinephrine concentration following the ingestion of 6 mg caffeine per kg body mass. The other six tetraplegic individuals did not show any increase in epinephrine and norepinephrine concentration. Another study [23] conducted with tetraplegic participants did not show a significant increase in plasma epinephrine and norepinephrine concentration following the ingestion of caffeine. Nevertheless, Van Soeren, Mohr [23] were able to detect a significant increase in plasma caffeine concentration with a peak concentration after 40 min. These results are in line with our own findings where we have found a significant increase in plasma caffeine concentration in tetraplegic participants but no increase in epinephrine and norepinephrine. It seems evident, that the ability to increase epinephrine and norepinephrine concentration depends on the level of lesion which influences sympathetic nervous system activity. Steinberg, Lauro [46] showed a significant increase in epinephrine and norepinephrine concentration from pre to post exercise performance in low lesioned paraplegic participants whereas high lesioned paraplegic participants showed only an increase in norepinephrine concentration. Schmid, Huonker [47] examined the same parameters in tetraplegic participants (even higher lesioned than high lesioned paraplegic participants). They found lower epinephrine and norepinephrine concentrations at rest in tetraplegic compared to able-bodied participants. In addition, they did not find any significant increase in epinephrine and norepinephrine concentrations from rest to maximal exercise performance in tetraplegic participants. These studies showed a diminished function of the sympathetic nervous system dependent of the lesion level in case of a spinal cord injury. This is very important when it comes to caffeine supplementation in order to increase sympathetic nervous system activity. This hypothesis is supported by findings of Flueck, Lienert [30] whereas the ingestion of caffeine in a group of tetraplegic individuals showed no ergogenic effect. In summary, it is very important to know, whether these participants are able to increase sympathetic modulation through caffeine consumption and to what extent.

### Limitations

Respiration seemed to influence HRV [48], which could confound our study results. We standardized breathing through metronomic breathing strategy but we did not standardize tidal volume. Through the ingestion of caffeine, tidal volume was increased, which would influence HRV. Nevertheless, HRV was shown to be reproducible in spinal cord injured participants [12], therefore, we assume our measures to be reliable. However, HF seemed to be influenced by a change in tidal volume [33, 48] and maybe that’s the reason why this parameter was not reliable in spinal cord injured participants [12]. In addition, all our participants were regular caffeine users who were limited in caffeine consume 12 hours before the trials. Maybe, we should have restricted caffeine consume the last week before the trials or should have chosen caffeine nonusers, which would have been a challenge in this specific group of participants. Thus, there would be no need to discuss the reason for an increase in HF in the placebo trial compared to the caffeine trial.

## Conclusion

To conclude, LF/HF seemed to be decreased after caffeine ingestion in all of our participants with a significant decrease in the paraplegic group. Systolic and diastolic blood pressure, as well as tidal volume increased following the ingestion of caffeine with a significant decrease in heart rate in both, placebo and caffeine, trials. Able-bodied and paraplegic participants were able to increase epinephrine concentrations even though all of our participants showed an increase in plasma caffeine concentrations. The influence of caffeine on the autonomic nervous system seems to depend on the level of lesion and the extent of the impairment. Therefore, tetraplegic participants may be less influenced by caffeine and they would probably benefit less in terms of activating the sympathetic nervous system.

## Supporting Information

S1 File Consort Checklist. (DOCX)

S2 File Clinical Trial Protocol. (DOCX)

## References

[1] Krassioukov A, West C. The role of autonomic function on sport performance in athletes with spinal cord injury. PM R. 2014;6(8 Suppl):S58–65. Epub 2014/08/20. 10.1016/j.pmrj.2014.05.023 . 25134753

[2] Schantz P, Sjoberg B, Widebeck AM, Ekblom B. Skeletal muscle of trained and untrained paraplegics and tetraplegics. Acta Physiol Scand. 1997;161(1):31–9. Epub 1997/10/06. 10.1046/j.1365-201X.1997.201371000.x 9381947

[3] Biering-Sorensen F, Bohr HH, Schaadt OP. Longitudinal study of bone mineral content in the lumbar spine, the forearm and the lower extremities after spinal cord injury. Eur J Clin Invest. 1990;20(3):330–5. Epub 1990/06/01. . 2114994 10.1111/j.1365-2362.1990.tb01865.x

[4] Cruz CD, Cruz F. Spinal cord injury and bladder dysfunction: new ideas about an old problem. Scie World J. 2011;11:214–34. Epub 2011/01/25. 10.1100/tsw.2011.26 . 21258763 PMC5720001

[5] Fynne L, Worsoe J, Gregersen T, Schlageter V, Laurberg S, Krogh K. Gastric and small intestinal dysfunction in spinal cord injury patients. Acta Neurol Scand. 2012;125(2):123–8. Epub 2011/03/25. 10.1111/j.1600-0404.2011.01508.x . 21428967

[6] Krogh K, Mosdal C, Laurberg S. Gastrointestinal and segmental colonic transit times in patients with acute and chronic spinal cord lesions. Spinal Cord. 2000;38(10):615–21. Epub 2000/11/28. . 11093323 10.1038/sj.sc.3101066

[7] Currie KD, West CR, Hubli M, Gee CM, Krassioukov AV. Peak heart rates and sympathetic function in tetraplegic nonathletes and athletes. Med Sci Sports Exerc. 2015;47(6):1259–64. Epub 2014/09/12. 10.1249/MSS.0000000000000514 . 25211366

[8] Billman GE. The LF/HF ratio does not accurately measure cardiac sympatho-vagal balance. Frontiers in physiology. 2013;4:26 Epub 2013/02/23. 10.3389/fphys.2013.00026 23431279 PMC3576706

[9] Carter JB, Banister EW, Blaber AP. The effect of age and gender on heart rate variability after endurance training. Med Sci Sports Exerc. 2003;35(8):1333–40. Epub 2003/08/06. 10.1249/01.MSS.0000079046.01763.8F . 12900687

[10] Furlan R, Porta A, Costa F, Tank J, Baker L, Schiavi R, et al Oscillatory patterns in sympathetic neural discharge and cardiovascular variables during orthostatic stimulus. Circulation. 2000;101(8):886–92. Epub 2000/03/01. . 10694528 10.1161/01.cir.101.8.886

[11] Montano N, Ruscone TG, Porta A, Lombardi F, Pagani M, Malliani A. Power spectrum analysis of heart rate variability to assess the changes in sympathovagal balance during graded orthostatic tilt. Circulation. 1994;90(4):1826–31. Epub 1994/10/01. . 7923668 10.1161/01.cir.90.4.1826

[12] Ditor DS, Kamath MV, Macdonald MJ, Bugaresti J, McCartney N, Hicks AL. Reproducibility of heart rate variability and blood pressure variability in individuals with spinal cord injury. Clin Auton Res. 2005;15(6):387–93. Epub 2005/12/20. 10.1007/s10286-005-0293-4 . 16362541

[13] Bunten DC, Warner AL, Brunnemann SR, Segal JL. Heart rate variability is altered following spinal cord injury. Clin Auton Res. 1998;8(6):329–34. Epub 1998/12/30. . 9869550 10.1007/BF02309623

[14] Agiovlasitis S, Heffernan KS, Jae SY, Ranadive SM, Lee M, Mojtahedi MC, et al Effects of paraplegia on cardiac autonomic regulation during static exercise. Am J Phys Med Rehabil. 2010;89(10):817–23. Epub 2010/09/22. 10.1097/PHM.0b013e3181f1b6e7 . 20855982

[15] Zamuner AR, Silva E, Teodori RM, Catai AM, Moreno MA. Autonomic modulation of heart rate in paraplegic wheelchair basketball players: Linear and nonlinear analysis. J Sports Sci. 2013;31(4):396–404. Epub 2012/10/24. 10.1080/02640414.2012.734917 . 23088300

[16] Takahashi M, Matsukawa K, Nakamoto T, Tsuchimochi H, Sakaguchi A, Kawaguchi K, et al Control of heart rate variability by cardiac parasympathetic nerve activity during voluntary static exercise in humans with tetraplegia. J Appl Physiol (1985). 2007;103(5):1669–77. Epub 2007/09/01. 10.1152/japplphysiol.00503.2007 . 17761788

[17] Wecht JM, De Meersman RE, Weir JP, Spungen AM, Bauman WA. Cardiac autonomic responses to progressive head-up tilt in individuals with paraplegia. Clin Auton Res. 2003;13(6):433–8. Epub 2003/12/16. 10.1007/s10286-003-0115-5 . 14673693

[18] Koh J, Brown TE, Beightol LA, Ha CY, Eckberg DL. Human autonomic rhythms: vagal cardiac mechanisms in tetraplegic subjects. J Physiol. 1994;474(3):483–95. Epub 1994/02/01. 8014908 10.1113/jphysiol.1994.sp020039 PMC1160339

[19] Castiglioni P, Di Rienzo M, Veicsteinas A, Parati G, Merati G. Mechanisms of blood pressure and heart rate variability: an insight from low-level paraplegia. American journal of physiology Regulatory, integrative and comparative physiology. 2007;292(4):R1502–9. Epub 2006/11/24. 10.1152/ajpregu.00273.2006 . 17122332

[20] Grimm DR, De Meersman RE, Almenoff PL, Spungen AM, Bauman WA. Sympathovagal balance of the heart in subjects with spinal cord injury. Am J Physiol. 1997;272(2 Pt 2):H835–42. Epub 1997/02/01. . 9124446 10.1152/ajpheart.1997.272.2.H835

[21] Teasell RW, Arnold JM, Krassioukov A, Delaney GA. Cardiovascular consequences of loss of supraspinal control of the sympathetic nervous system after spinal cord injury. Arch Phys Med Rehabil. 2000;81(4):506–16. Epub 2000/04/18. 10.1053/mr.2000.3848 . 10768544

[22] Inoue K, Ogata H, Hayano J, Miyake S, Kamada T, Kuno M, et al Assessment of autonomic function in traumatic quadriplegic and paraplegic patients by spectral analysis of heart rate variability. J Auton Nerv Syst. 1995;54(3):225–34. Epub 1995/09/05. . 7490424 10.1016/0165-1838(95)00012-m

[23] Van Soeren M, Mohr T, Kjaer M, Graham TE. Acute effects of caffeine ingestion at rest in humans with impaired epinephrine responses. J Appl Physiol. 1996;80(3):999–1005. Epub 1996/03/01. . 8964766 10.1152/jappl.1996.80.3.999

[24] Van Soeren MH, Sathasivam P, Spriet LL, Graham TE. Caffeine metabolism and epinephrine responses during exercise in users and nonusers. J Appl Physiol. 1993;75(2):805–12. Epub 1993/08/01. . 8226485 10.1152/jappl.1993.75.2.805

[25] Tarnopolsky MA. Caffeine and creatine use in sport. Ann Nutr Metab. 2010;57 Suppl 2:1–8. Epub 2010/01/01. 000322696 [pii] 10.1159/000322696 . 21346331

[26] Tarnopolsky MA. Caffeine and endurance performance. Sports Med. 1994;18(2):109–25. Epub 1994/08/01. . 9132918 10.2165/00007256-199418020-00004

[27] Burke LM. Caffeine and sports performance. Appl Physiol Nutr Metab. 2008;33(6):1319–34. Epub 2008/12/18. h08-130 [pii] 10.1139/H08-130 . 19088794

[28] Quintana DS, Heathers JA. Considerations in the assessment of heart rate variability in biobehavioral research. Frontiers in psychology. 2014;5:805 Epub 2014/08/08. 10.3389/fpsyg.2014.00805 25101047 PMC4106423

[29] Kivity S, Ben Aharon Y, Man A, Topilsky M. The effect of caffeine on exercise-induced bronchoconstriction. Chest. 1990;97(5):1083–5. Epub 1990/05/01. . 2184994 10.1378/chest.97.5.1083

[30] Flueck JL, Lienert M, Schaufelberger F, Krebs J, Perret C. Ergogenic Effects of Caffeine Consumption in a 3 min All-Out Arm Crank Test in Paraplegic and Tetraplegic Compared to Able-Bodied Individuals. Int J Sport Nutr Exerc Metab. 2015 Epub 2015/07/02. 10.1123/ijsnem.2015-0090 . 26132642

[31] Tarvainen MP, Ranta-Aho PO, Karjalainen PA. An advanced detrending method with application to HRV analysis. IEEE Trans Biomed Eng. 2002;49(2):172–5. Epub 2002/06/18. 10.1109/10.979357 . 12066885

[32] Burr RL. Interpretation of normalized spectral heart rate variability indices in sleep research: a critical review. Sleep. 2007;30(7):913–9. Epub 2007/08/09. 17682663 10.1093/sleep/30.7.913 PMC1978375

[33] Hibino G, Moritani T, Kawada T, Fushiki T. Caffeine enhances modulation of parasympathetic nerve activity in humans: quantification using power spectral analysis. J Nutr. 1997;127(7):1422–7. Epub 1997/07/01. . 9202101 10.1093/jn/127.7.1422

[34] Monda M, Viggiano A, Vicidomini C, Iannaccone T, Tafuri D, De Luca B. Espresso coffee increases parasympathetic activity in young, healthy people. Nutr Neurosci. 2009;12(1):43–8. Epub 2009/01/31. 10.1179/147683009X388841 . 19178791

[35] Notarius CF, Floras JS. Caffeine Enhances Heart Rate Variability in Middle-Aged Healthy, But Not Heart Failure Subjects. Journal of caffeine research. 2012;2(2):77–82. Epub 2012/06/01. 10.1089/jcr.2012.0010 24761268 PMC3621323

[36] Richardson T, Rozkovec A, Thomas P, Ryder J, Meckes C, Kerr D. Influence of caffeine on heart rate variability in patients with long-standing type 1 diabetes. Diabetes Care. 2004;27(5):1127–31. Epub 2004/04/28. . 15111532 10.2337/diacare.27.5.1127

[37] Rauh R, Burkert M, Siepmann M, Mueck-Weymann M. Acute effects of caffeine on heart rate variability in habitual caffeine consumers. Clin Physiol Funct Imaging. 2006;26(3):163–6. Epub 2006/04/28. 10.1111/j.1475-097X.2006.00663.x . 16640511

[38] Nurminen ML, Niittynen L, Korpela R, Vapaatalo H. Coffee, caffeine and blood pressure: a critical review. Eur J Clin Nutr. 1999;53(11):831–9. Epub 1999/11/11. . 10556993 10.1038/sj.ejcn.1600899

[39] Battram DS, Bugaresti J, Gusba J, Graham TE. Acute caffeine ingestion does not impair glucose tolerance in persons with tetraplegia. J Appl Physiol (1985). 2007;102(1):374–81. Epub 2006/10/28. 10.1152/japplphysiol.00901.2006 . 17068214

[40] Catz A, Mendelson L, Solzi P. Symptomatic postprandial hypotension in high paraplegia. Case report. Paraplegia. 1992;30(8):582–6. Epub 1992/08/01. 10.1038/sc.1992.118 . 1523000

[41] Graham-Paulson TS, Perret C, Watson P, Goosey-Tolfrey VL. Improvement of Sprint Performance in Wheelchair Sportsmen With Caffeine Supplementation. Int J Sports Physiol Perform. 2016;11(2):214–20. Epub 2015/07/17. 10.1123/ijspp.2015-0073 . 26182441

[42] Pianosi P, Grondin D, Desmond K, Coates AL, Aranda JV. Effect of caffeine on the ventilatory response to inhaled carbon dioxide. Respir Physiol. 1994;95(3):311–20. Epub 1994/03/01. . 8059074 10.1016/0034-5687(94)90093-0

[43] Kraaijenga JV, Hutten GJ, de Jongh FH, van Kaam AH. The effect of caffeine on diaphragmatic activity and tidal volume in preterm infants. J Pediatr Adolesc Gynecol. 2015;167(1):70–5. Epub 2015/05/20. 10.1016/j.jpeds.2015.04.040 . 25982138

[44] Fredholm BB. On the mechanism of action of theophylline and caffeine. Acta Med Scand. 1985;217(2):149–53. Epub 1985/01/01. . 2986418 10.1111/j.0954-6820.1985.tb01650.x

[45] Mohr T, Van Soeren M, Graham TE, Kjaer M. Caffeine ingestion and metabolic responses of tetraplegic humans during electrical cycling. J Appl Physiol. 1998;85(3):979–85. Epub 1998/09/08. . 9729573 10.1152/jappl.1998.85.3.979

[46] Steinberg LL, Lauro FA, Sposito MM, Tufik S, Mello MT, Naffah-Mazzacoratti MG, et al Catecholamine response to exercise in individuals with different levels of paraplegia. Braz J Med Biol Res. 2000;33(8):913–8. Epub 2000/08/02. . 10920433 10.1590/s0100-879x2000000800007

[47] Schmid A, Huonker M, Stahl F, Barturen JM, Konig D, Heim M, et al Free plasma catecholamines in spinal cord injured persons with different injury levels at rest and during exercise. J Auton Nerv Syst. 1998;68(1–2):96–100. Epub 1998/04/08. . 9531449 10.1016/s0165-1838(97)00127-6

[48] Poyhonen M, Syvaoja S, Hartikainen J, Ruokonen E, Takala J. The effect of carbon dioxide, respiratory rate and tidal volume on human heart rate variability. Acta Anaesthesiol Scand. 2004;48(1):93–101. Epub 2003/12/17. . 14674979 10.1111/j.1399-6576.2004.00272.x

