# Supplementary material for: Acute Effects of Caffeine on Heart Rate Variability, Blood Pressure and Tidal Volume in Paraplegic and Tetraplegic Compared to Able-Bodied Individuals: A Randomized, Blinded Trial
Source: PLoS One. 2016 Oct 24;11(10):e0165034. doi: 10.1371/journal.pone.0165034 (PMC5077167; doi:10.1371/journal.pone.0165034)
Supplement: S2 File — (DOCX) [file pone.0165034.s002.docx]

Clinical Study Protocol (Study Title) Study Identifier

This document is the xx Clinical Protocol template for IIT (Investigator initiated Trials) studies. XX strongly recommends using this template to develop clinical research protocols for trials testing an investigational medicinal product (IMP) or a medical device (MD) to be submitted to Swiss authorities.

This template is suitable for studies:

- involving IIT,
- performed in Switzerland, respectively where the Sponsor-Investigator is located in Switzerland
- where the study question does relate to the use of drug(s) or medical device effect(s),
- where the Swiss law on therapeutic products (HMG/LPTh and Federal Act on Medicinal Products and Medical Devices) applies,
- where the Swiss law on human research (LHR) and its applicable ordinance KlinV/OClin applies,
- that are interventional*

*health related interventional studies include research in preventive, diagnostic, therapeutic, palliative or rehabilitation activities that are examined in the context of a clinical trial.

The current template is based on:

- [AGEK – CT CER](http://www.swissethics.ch/templates.html) / [Swissmedic](http://www.swissmedic.ch/bewilligungen/00089/00282/index.html?lang=de) guidelines: “Studienprotokolle von klinischen‚ Investigator-initiated’ Studien/Versuchen / Exigences des protocoles d’études/d’essais cliniques initiés par l’investigateur” dated 24.02.2009,
- new law on human research ([LHR](http://www.bag.admin.ch/themen/medizin/00701/00702/07558/index.html?lang=de)) and its applicable ordinance [KlinV/OClin](http://www.bag.admin.ch/themen/medizin/00701/00702/12310/index.html?lang=de)
- the [SPIRIT statement](http://www.spirit-statement.org) and
- [ICH-GCP E6](http://www.ich.org/fileadmin/Public_Web_Site/ICH_Products/Guidelines/Efficacy/E6_R1/Step4/E6_R1__Guideline.pdf), section 6
- [EN ISO14155:2011](https://www.iso.org/obp/ui/#iso:std:iso:14155:ed-2:v1:en): Annex A
- Swiss clinical trial registration portal ([www.kofam.ch](http://www.kofam.ch)) - check name and link

This template attempts to provide a general format applicable to all clinical trials evaluating an investigational product (drugs or medical devices).

Note that *instructions* are indicated in *blue italics* and they need be deleted (or alternatively may be formatted as “hidden Text” that will not show in printing).

Section headings and template text formatted in **regular type red** gives you reference to the legal requirements. This text may be deleted.

Section headings and template text formatted in regular type (black) should be included in your protocol document as provided in the template.

Header and footer should contain the following information (on all pages): [Protocol Title], [Page x of xx], [version x, DD/MM/YYYY], [Study ID]

In places where the information is redundant, it is acceptable to reference another section, to document or to state its redundancy but the section has not to be deleted.

Refer questions regarding use of this protocol template to Claudio Perret, Sports Medicine, Swiss Paraplegic Centre Nottwil, Guido A. Zäch Strasse 4, 6207 Nottwil, +41 41 939 66 21, claudio.perret@paraplegie.ch

This template was developed by a task force initiated by the Federal Office of Public Health (FOPH) and the AGEK / CT CER during 2013 and under the lead and coordination of the Swiss Clinical Trial Organisation (SCTO), Basel. Clinical research experts from 8 institutions reviewed the template. The FOPH and AGEK reviewed the template and recommend its use.

Members of the task force no. 6 and contributors to this template:

- FOPH, Dr Andri Christen
- AGEK, Dr Wolfgang Tschacher
- CTC Zurich, Dr Eva Brombacher
- CTU St Gallen, Roger Getzmann
- CTC Lausanne and Cochrane Switzerland, Dr Erik von Elm
- CTC Geneva, Dr Christophe Combescure
- EOC Ticino, Dr Liliane Petrini
- SAKK, Dr Christiane Pilop
- SCTO, Annette Magnin and Dr Caecilia Schmid

Reviewers:

- EOC TI, Dr Claudio Gobbi
- EOC TI, Dr Mauro Manconi
- HUG: Prof. Dr Bernard Hirschel
- HUG / EC: Dr Sandrine Charvat
- Inselspital / Uni. Bern, Prof. Dr Peter Jüni, Dr Sven Trelle
- KSSG, Prof. Dr Christoph Driessen
- SAKK, Dr Pirus Ghadjar (Charité Berlin)
- SPZ / EC Luzern, Dr Angela Frotzler
- USZ, PD Dr Christian Baumann
- USZ, Dr Cédric Poyet
- Scienceindustries, Dr Daniela Gunz

<<Protocol template: Interventional study with investigational medicinal product (IMP) / medical device (MD)>>

Clinical Study Protocol

Influence of caffeine on heart rate variability and performance in paraplegic and tetraplegic subjects compared to able-bodied subjects: a pilot study

A placebo controlled, randomised and double-blind study conducted with disabled and abled-bodied healthy subjects

HRV and caffeine in disabled and able-bodied subjects

| Study Type: | Clinical trial |
| --- | --- |
| Study Categorisation: | Low risk A |
| Study Registration: | ClinicalTrials.gov NCT02083328 |
| Study Identifier: | - |
| Sponsor, Sponsor-Investigator or Principal Investigator: | Claudio Perret, Sports Medicine, Swiss Paraplegic Centre Nottwil  Guido A. Zäch Strasse 4, 6207 Nottwil, +41 41 939 66 21, claudio.perret@paraplegie.ch |
| Investigational Product: | Caffeine and Mannitol as a placebo |
| Protocol Version and Date: | Version 2, 15.07.2014 |

CONFIDENTIAL

“The information contained in this document is confidential and the property of the Swiss Paraplegic Centre in Nottwil. The information may not - in full or in part - be transmitted, reproduced, published, or disclosed to others than the applicable Competent Ethics Committee(s) and Regulatory Authority(ies) without prior written authorisation from the sponsor except to the extent necessary to obtain informed consent from those who will participate in the study.

Signature Page(s)

| Study number | kofam.ch: ID-nr. SNCTP000000874 |
| --- | --- |
| Study Title | Influence of caffeine on heart rate variability and performance in paraplegic and tetraplegic subjects compared to able-bodied subjects: a pilot study |

The Sponsor-Investigator and trial statistician have approved the protocol version 2 (dated 15.07.14), and confirm hereby to conduct the study according to the protocol, current version of the World Medical Association Declaration of Helsinki, ICH-GCP guidelines or ISO 14155 norm if applicable and the local legally applicable requirements.

Sponsor-Investigator:

| Place/Date |  | Signature |
| --- | --- | --- |

Table of Contents

Study synopsis 8

study summary in local language *(German)* 10

Abbreviations 11

Study schedule 12

1. STUDY ADMINISTRATIVE STRUCTURE 13

1.1 Sponsor, Sponsor-Investigator 13

1.2 Principal Investigator(s) 13

1.3 Statistician ("Biostatistician") 13

1.4 Laboratory 13

1.5 Monitoring institution 13

1.6 Data Safety Monitoring Committee 13

1.7 Any other relevant Committee, Person, Organisation, Institution 13

2. ETHICAL AND REGULATORY ASPECTS 14

2.1 Study registration 14

2.2 Categorisation of study (revise text once English translation is provided by BAG!) 14

2.3 Competent Ethics Committee (CEC) 14

2.4 Competent Authorities (CA) 14

2.5 Ethical Conduct of the Study 14

2.6 Declaration of interest 14

2.7 Patient Information and Informed Consent 14

2.8 Participant privacy and confidentiality 15

2.9 Early termination of the study 15

2.10 Protocol amendments 15

3. Background and Rationale 16

3.1 Background and Rationale 16

3.2 Investigational Product (treatment, device) and Indication 16

3.3 Preclinical Evidence 17

3.4 Clinical Evidence to Date 17

3.5 Dose Rationale / Medical Device: Rationale for the intended purpose in study (pre-market MD) 17

3.6 Explanation for choice of comparator (or placebo) 18

3.7 Risks / Benefits 18

3.8 Justification of choice of study population 18

4. STUDY OBJECTIVES 19

4.1 Overall Objective 19

4.2 Primary Objective 19

4.3 Secondary Objectives 19

4.4 Safety Objectives 19

5. STUDY OUTCOMES 20

5.1 Primary Outcome 20

5.2 Secondary Outcomes 20

5.3 Other Outcomes of Interest 20

5.4 Safety Outcomes 20

6. STUDY DESIGN 21

6.1 General study design and justification of design 21

6.2 Methods of minimising bias 22

6.2.1 Randomisation 22

6.2.2 Blinding procedures 23

6.2.3 Other methods of minimising bias 23

6.3 Unblinding Procedures (Code break) 23

7. STUDY POPULATION 24

7.1 Eligibility criteria 24

7.2 Recruitment and screening 24

7.3 Assignment to study groups 24

7.4 Criteria for withdrawal / discontinuation of participants 24

8. STUDY INTERVENTION 25

8.1 Identity of Investigational Products (treatment / medical device) 25

8.1.1 Experimental Intervention (treatment / medical device) 25

8.1.2 Control Intervention (standard/routine/comparator treatment / medical device) 25

8.1.3 Packaging, Labelling and Supply (re-supply) 25

8.1.4 Storage Conditions 25

8.2 Administration of experimental and control interventions 25

8.2.1 Experimental Intervention 25

8.2.2 Control Intervention 25

8.3 Dose / Device modifications 25

8.4 Compliance with study intervention 26

8.5 Data Collection and Follow-up for withdrawn participants 26

8.6 Trial specific preventive measures 26

8.7 Concomitant Interventions (treatments) 26

8.8 Study Drug / Medical Device Accountability 26

8.9 Return or Destruction of Study Drug / Medical Device 26

9. STUDY ASSESSMENTS 27

9.1 Study flow chart(s) / table of study procedures and assessments 27

9.2 Assessments of outcomes 27

9.2.1 Assessment of primary outcome 27

9.2.2 Assessment of secondary outcomes 27

9.2.3 Assessment of other outcomes of interest 28

9.2.4 Assessment of safety outcomes 28

9.2.5 Assessments in participants who prematurely stop the study 29

9.3 Procedures at each visit 29

9.3.1 Split into subtitles by type of visit 29

9.3.2 Split into subtitles by type of visit 29

10. SAFETY 31

10.1 Drug studies 31

10.1.1 Definition and assessment of (serious) adverse events and other safety related events 31

10.1.2 Reporting of serious adverse events (SAE) and other safety related events 32

10.1.3 Follow up of (Serious) Adverse Events 32

11. STATISTICAL METHODS 33

11.1 Hypothesis 33

11.2 Determination of Sample Size 33

11.3 Statistical criteria of termination of trial 33

11.4 Planned Analyses 33

11.4.1 Datasets to be analysed, analysis populations 33

11.4.2 Primary Analysis 33

11.4.3 Secondary Analyses 34

11.4.4 Interim analyses 34

11.4.5 Safety analysis 34

11.4.6 Deviation(s) from the original statistical plan 34

11.5 Handling of missing data and drop-outs 34

12. QUALITY ASSURANCE AND CONTROL 35

12.1 Data handling and record keeping / archiving 35

12.1.1 Case Report Forms 35

12.1.2 Specification of source documents 35

12.1.3 Record keeping / archiving 35

12.2 Data management 35

12.2.1 Data Management System 35

12.2.2 Data security, access and back-up 36

12.2.3 Analysis and archiving 36

12.2.4 Electronic and central data validation 36

12.3 Monitoring 36

12.4 Audits and Inspections 36

12.5 Confidentiality, Data Protection 36

12.6 Storage of biological material and related health data 36

13. PUBLICATION AND DISSEMINATION POLICY 37

14. FUNDING AND SUPPORT 38

14.1 Funding 38

14.2 Other Support 38

15. INSURANCE 38

16. REFERENCES 39

17. APPENDICES 41

Study synopsis

| Sponsor / Sponsor-Investigator | Swiss Paraplegic Centre Nottwil / PD Dr. sc. nat. Claudio Perret |
| --- | --- |
| Study Title: | Influence of caffeine on heart rate variability and performance in paraplegic and tetraplegic subjects compared to able-bodied subjects: a pilot study |
| Short Title / Study ID: | HRV and caffeine in disabled and able-bodied subjects. |
| Protocol Version and Date: | Version No. 2, 15.07.2014 |
| Trial registration: | ClinicalTrials.gov NCT02083328 |
| Study category and Rationale | Low Risk Clinical Trial (Category A) (Supplementation with caffeine corresponds to international guidelines) |
| Clinical Phase: | N/A |
| Background and Rationale: | The aim is to investigate the influence of caffeine on heart rate variability and on performance in a 3 min exercise test in different population groups (able-bodied, paraplegic and tetraplegic subjects). |
| Objective(s): | In general, we are interested in the differences between the heart rate variability before and after caffeine supplementation and if exercise performance is enhanced using this type of supplementation. Another objective is to see whether there are differences between abled-bodied and disabled subjects as subjects with spinal cord injury have an impairment of the autonomic nervous system. |
| Outcome(s): | Primary outcome parameters are differences in heart rate variability and power output in the exercise performance test between caffeine and placebo trial. Secondary outcome parameters are epinephrine and norepinephrine concentration before and after caffeine supplementation, heart rate, tidal volume, lactate concentration before and after the exercise testing, as well as rating of perceived exertion after the exercise performance test. |
| Study design: | Randomised, double-blind, placebo controlled trial (RCT); pilot study |
| Inclusion / Exclusion criteria: | Inclusion: healthy, non-smoker, minimal 3x45 min physical activity per week, tetraplegic (C5 to C7) or paraplegic (sub Th8), AIS A, termination of first rehabilitation and more than 6 months after trauma.  Exclusion: drugs or supplements which influence heart rate variability, diabetes |
| Measurements and procedures: | Heart rate variability (HRV) is measured using a Polar heart rate monitor. After a 10min rest, HRV is measured during a 5 min phase in supine position and afterwards during a 6 min time period in sitting position. These measurements are conducted before the ingestion of the supplement, before the exercise performance test and after the exercise performance test.  Epinephrine and norepinephrine concentrations are measured before supplementation and one hour after supplementation (venous blood withdrawal).  Resting tidal volume is measured during the measurements of HRV.  Power output during the 3 min exercise performance test is analysed.  Lactate concentration is measured before and after the exercise performance testing taking a capillary blood sample.  Blood pressure is measured immediately before every HRV measurement.  Rate of perceived exertion is measured after the warm up and after the exercise performance test. |
| Study Product / Intervention: | Caffeine: 6 mg/kg body mass dosage, supplement will be ingested 1 hour before the exercise performance test respectively 45 min before the HRV measurement. |
| Control Intervention (if applicable): | Placebo (mannitol) |
| Number of Participants with Rationale: | 30 participants in total (10 per each group of able-bodied, tetraplegic and paraplegic subjects): this study is a pilot study thus no power analysis was performed. |
| Study Duration: | 20 months |
| Study Schedule: | 01. May 2014: start of data collection  31. December 2015: end of data collection |
| Investigator(s): | PD Dr. sc. nat. Claudio Perret  Sports Medicine, Swiss Paraplegic Centre, Guido A. Zäch Strasse 4, 6207 Nottwil, +41 41 939 66 21, claudio.perret@paraplegie.ch |
| Study Centre(s): | Single-center study: Swiss Paraplegic Centre Nottwil |
| Statistical Considerations: | For analysing the differences pre and post ingestion of caffeine within a subject, a Wilcoxon test will be used.  The correlation between HRV and performance under caffeine treatment will be evaluated with the Spearman’s rank correlation coefficient. |
| GCP Statement: | This study will be conducted in compliance with the protocol, the current version of the Declaration of Helsinki, the ICH-GCP or ISO EN 14155 (as far as applicable) as well as all national legal and regulatory requirements. |

study summary in local language can be provided here (German)

Die Herzfrequenzvariabilität (HRV) beschreibt die Varianz der Zeitintervalle zwischen den einzelnen Herzschlägen. Sie ist ein Parameter zur Bestimmung der Funktion des Sympathikus und Parasympathikus des autonomen Nervensystems. Diese beiden Komponenten haben antagonistische Aufgaben bei der Regulation der viszeralen Funktion des Körpers. Während der Sympathikus eine eher anregende Wirkung hat, hemmt der Parasympathikus.

Aus der Analyse der HRV resultieren zwei Komponenten, welche den zwei Systemen zugeordnet werden können. Die low frequency (LF) repräsentiert den Sympathikus während die high frequency (HF) dem Parasympathikus zugeordnet wird.

Je mehr also zum Beispiel der Sympathikus aktiviert ist, desto grösser ist der Anteil des LF- im Vergleich zur HF-Komponente. Mit der Analyse der HRV lassen sich so Aussagen über die sympathische-parasympathische Balance machen.

Bei querschnittgelähmten Personen ist durch die Verletzung des Rückenmarks auch das autonome Nervensystem betroffen. Davon ist, aufgrund seiner Austritthöhe entlang der Wirbelsäule bis ca. zum 6. Brustwirbel, nur der sympathische Anteil betroffen. Der Parasympathikus dagegen tritt auf Hirnstamm-Höhe aus und ist so nicht direkt von der Unterbrechung der Rückenmarks betroffen.

Je höher die Läsion, desto höher ist die Beeinträchtigung des Sympathikus. Dies hat folglich auch Auswirkungen auf die HRV: Das sympathische-parasympathische Gleichgewicht ist aufgrund des Sympathikus-Ausfalls gestört. So haben HRV-Messungen bei Tetraplegikern ergeben, dass die LF-Komponente meist nicht mehr vorhanden ist und gleichzeitig die HF-Komponente erniedrigt ist. Bei Paraplegikern wurde zwar die LF und die HF gemessen, jedoch waren beide Komponenten abgeschwächt im Vergleich zu gesunden Referenzpersonen.

Welche Auswirkungen ein Stimulanz des autonomen Nervensystems auf die HRV bei Querschnittgelähmten hat, wurde bisher noch nicht untersucht. Wir nehmen an, dass eine Supplementierung mit Coffein messbare Effekte auf die HRV bei dieser Patientengruppe hat. Bei gesunden Referenzpersonen wurde durch eine Supplementierung mit Coffein eine Leistungsverbesserung im anaeroben hochintensiven Bereich beobachtet. Ob dieser Effekt auch bei Querschnittgelähmten auftritt, ist noch nicht abschliessend geklärt.

Mit unserer Studie untersuchen wir den Effekt einer Coffeinsupplementierung auf die HRV und die Leistungsfähigkeit bei querschnittgelähmten Probanden im Vergleich zu gesunden Referenzpersonen.

Dabei vergleichen wir die HRV-Werte vor und nach Einnahme von Coffein innerhalb und zwischen den Gruppen (Tetraplegiker, Paraplegiker, Referenzpersonen) und messen den Effekt auf die Leistungsfähigkeit durch eine Coffeinsupplementierung.

Durch diese Studie möchten wir mehr Grundlagenwissen im Bereich der HRV-Messung bei Querschnittgelähmten erlangen, um so die Möglichkeit der Verwendung einer solchen Messung im Bereich der Klassifizierung der Läsionen zu untersuchen oder beim Einsatz der Trainingsgestaltung bzw. der Vorbeugung von Übertraining. Weiter erhoffen wir uns durch die Studie mehr Kenntnisse über die Sinnhaftigkeit einer Coffeinsupplementierung bei querschnittgelähmten Athleten.

Die geplante Studie soll neue Erkenntnisse erschliessen und so einen Schritt zum besseren Verständnis der Funktionsweise der HRV bei Querschnittgelähmten beitragen.

Abbreviations

| AE | Adverse Event |
| --- | --- |
| ANS  CA | Autonomic Nervous System  Competent Authority (e.g. Swissmedic) |
| CEC | Competent Ethics Committee |
| CRF | Case Report Form |
| eCRF | Electronic Case Report Form |
| CTCAE | Common terminology criteria for adverse events |
| DSUR | Development safety update report |
| GCP | Good Clinical Practice |
| IB | Investigator’s Brochure |
| Ho | Null hypothesis |
| H1 | Alternative hypothesis |
| HFG | Humanforschungsgesetz (Law on human research) |
| HMG | Heilmittelgesetz |
| HRV  IMP | Heart rate variability  Investigational Medicinal Product |
| IIT | Investigator-initiated Trial |
| ISO | International Organisation for Standardisation |
| ITT | Intention to treat |
| KlinV | Verordnung über klinische Versuche in der Humanforschung |
| LHR | Law on human research |
| LPTh | Loi sur les produits thérapeutiques |
| LRH | Loi fédérale relative à la recherche sur l’être humain |
| MD | Medical Device |
| OClin | Ordonnance sur les essais cliniques dans le cadre de la recherche sur l'être humain |
| PI  RPE | Principal Investigator  Rate of perceived exertion |
| SDV  SCI | Source Data Verification  Spinal Cord Injury |
| SOP | Standard Operating Procedure |
| SPC | Summary of product characteristics |
| SUSAR | Suspected Unexpected Serious Adverse Reaction |
| TMF | Trial Master File |

Study schedule

| Study Periods | Recruitment | Screening and Familiarization | Intervention Period | |
| --- | --- | --- | --- | --- |
| Visit | 1 | 2 | 3 | 4 |
| Time | individual | individual | 09:00 | 09:00 |
| Patient Information and Informed Consent | X | - | - | - |
| Subject Group | X | X | X | X |
| Medical History | X | - | - | - |
| In- /Exclusion Criteria | X | - | - | - |
| Checklist | - | - | X | X |
| VO_2max_ Test | - | X | - | - |
| Vital Signs | - | - | X | X |
| Laboratory Tests (Catecholamine) | - | - | X | X |
| Randomisation | X | - | - | - |
| HRV Measurement | - | - | X | X |
| 3 min Performance Test | - | X | X | X |
| Tidal Volume Measurement | - | - | X | X |
| Venous Blood Sample | - | - | X | X |
| Capillary Blood Sample | - | - | X | X |
| Blood Glucose Level | X | - | - | - |
| Blood Pressure Measurement | - | - | X | X |
| Ingestion of Caffeine or Placebo | - | - | X | X |
| Adverse Events | - | - | X | X |

# STUDY ADMINISTRATIVE STRUCTURE

## Sponsor, Sponsor-Investigator

Swiss Paraplegic Centre Nottwil, Guido A. Zäch Strasse 1, 6207 Nottwil

## Principal Investigator(s)

PD Dr. sc. nat. Claudio Perret, Sports Medicine, Swiss Paraplegic Centre, Guido A. Zäch Strasse 4, 6207 Nottwil, +41 41 939 66 21, [claudio.perret@paraplegie.ch](mailto:claudio.perret@paraplegie.ch)

## Statistician ("Biostatistician")

Dr. med. vet. Jörg Krebs, Clinical Trial Unit, Guido A. Zäch Strasse 4, 6207 Nottwil, [joerg.krebs@paraplegie.ch](mailto:joerg.krebs@paraplegie.ch)

## Laboratory

The venous blood samples will be analysed by Synlab Luzern, Alpenquai 14, 6002 Luzern, +41 41 360 35 35

## Monitoring institution

Clinical Trial Unit, Guido A. Zäch Strasse 4, 6207 Nottwil, +41 41 939 55 63, [clinical.trial.unit@paraplegie.ch](mailto:clinical.trial.unit@paraplegie.ch)

## Data Safety Monitoring Committee

Clinical Trial Unit, Guido A. Zäch Strasse 4, 6207 Nottwil, [clinical.trial.unit@paraplegie.ch](mailto:clinical.trial.unit@paraplegie.ch)

The Clinical Trial Unit of the Swiss Paraplegic Centre in Nottwil is independent of the Institute of Sports Medicine in Nottwil. They provide us with the web-based data capture software secuTrial and manage the access to data input and storage.

## Any other relevant Committee, Person, Organisation, Institution

MD Matthias Strupler, Head of Sports Medicine, Guido A. Zäch Strasse 4, 6207 Nottwil, [matthias.strupler@paraplegie.ch](mailto:matthias.strupler@paraplegie.ch), +41 41 939 66 11

MSc Joëlle Flück, PhD Student and study coordination, Guido A. Zäch Strasse 4, 6207 Nottwil, [joelle.flueck@paraplegie.ch](mailto:joelle.flueck@paraplegie.ch), +41 41 939 66 17

BSc Fabienne Schaufelberger, Master Student, Guido A. Zäch Strasse 4, 6207 Nottwil, [fabienne.schaufelberger@paraplegie.ch](mailto:fabienne.schaufelberger@paraplegie.ch)+41 41 939 66 15,

Msc Valentin Habermacher, Pharmacist, Swiss Paraplegic Centre Nottwil, Guido A. Zäch Strasse 1, 6207 Nottwil, [valentin.habermacher@paraplegie.ch](mailto:valentin.habermacher@paraplegie.ch), +41 41 939 59 56

PD MD Matthias Wilhelm, Head Physician, Cardiovascular Prevention, Rehabilitation and Sports Medicine Inselspital Bern, Anna-Seiler-Haus Inselspital, 3010 Bern, [matthias.wilhelm@insel.ch](mailto:matthias.wilhelm@insel.ch)

Msc Daniela Schäfer; PhD Student, Cardiovascular Prevention, Rehabilitation and Sports Medicine Inselspital Bern, Anna-Seiler-Haus Inselspital, 3010 Bern, [daniela.schaefer@insel.ch](mailto:daniela.schaefer@insel.ch)

# ETHICAL AND REGULATORY ASPECTS

The decision of the CEC concerning the conduct of the study will be made in writing to the Sponsor-Investigator before commencement of this study. The clinical study can only begin once approval from all required authorities has been received. Any additional requirements imposed by the authorities shall be implemented.

## Study registration

The study is registered on clinicaltrials.gov with the registration number NCT 02083328. Furthermore the study is registered on kofam.ch (kofam-ID-nr. SNCTP000000874).

## Categorisation of study (revise text once English translation is provided by BAG!)

Our study is a clinical trial with the supplement caffeine. It takes part in the Category A of low risk trials as our ingested caffeine dose corresponds to the international guidelines for caffeine supplementation in exercise performance.

## Competent Ethics Committee (CEC)

The study was presented at the internal scientific study committee (EGKF) at the Swiss Paraplegic Centre in Nottwil and the scientificity and ethical considerations were approved.

No changes are made to the protocol without prior Ethic Committee and Sponsor approval, except where necessary to eliminate apparent immediate hazards to study participants.

Premature study end or interruption of the study is reported within 15 days. The regular end of the study is reported to the CEC within 90 days, the final study report shall be submitted within one year after study end. Amendments are reported according to chapter 3.10.

## Competent Authorities (CA)

Not necessary since study of category A (low risk).

## Ethical Conduct of the Study

The study will be carried out in accordance to the protocol and with principles enunciated in the current version of the Declaration of Helsinki, the guidelines of Good Clinical Practice (GCP) issued by ICH, in case of medical device: the European Directive on medical devices 93/42/EEC and the ISO Norm 14155 and ISO 14971, the Swiss Law and Swiss regulatory authority’s requirements. The CEC and regulatory authorities will receive annual safety and interim reports and be informed about study stop/end in agreement with local requirements.

Add other local requirements in case of medical device or international studies.

## Declaration of interest

The principal investigator declares that there is no conflict of interest, neither intellectual nor financial or proprietary conflict of interest.

## Patient Information and Informed Consent

The investigators will explain to each participant the nature of the study, its purpose, the procedures involved, the expected duration, the potential risks and benefits and any discomfort it may entail. Each participant will be informed that the participation in the study is voluntary and that he may withdraw from the study at any time and that withdrawal of consent will not affect his subsequent medical assistance and treatment.

The participant must be informed that his medical records may be examined by authorised individuals other than their treating physician.

All participants for the study will be provided a participant information sheet and a consent form describing the study and providing sufficient information for participant to make an informed decision about their participation in the study. The subject will get this information one week before the start of their own testing phase. So, enough time will be provided to decide whether he will participate or not.

The patient information sheet and the consent form will be submitted to the CEC and to the competent authority (as applicable) to be reviewed and approved. The formal consent of a participant, using the approved consent form, must be obtained before the participant is submitted to any study procedure.

The participant should read and consider the statement before signing and dating the informed consent form, and should be given a copy of the signed document. The consent form must also be signed and dated by the investigator (or his designee) and it will be retained as part of the study records.

## Participant privacy and confidentiality

The investigator affirms and upholds the principle of the participant's right to privacy and that they shall comply with applicable privacy laws. Especially, anonymity of the participants shall be guaranteed when presenting the data at scientific meetings or publishing them in scientific journals.

Individual subject medical information obtained as a result of this study is considered confidential and disclosure to third parties is prohibited. Subject confidentiality will be further ensured by utilising subject identification code numbers to correspond to treatment data in the computer files.

For data verification purposes, authorised representatives of the Sponsor (-Investigator), a competent authority (e.g. Swissmedic), or an ethics committee may require direct access to parts of the medical records relevant to the study, including participants’ medical history.

## Early termination of the study

The Sponsor-Investigator may terminate the study prematurely according to certain circumstances, for example:

- ethical concerns,
- insufficient participant recruitment,
- when the safety of the participants is doubtful or at risk, respectively,
- alterations in accepted clinical practice that make the continuation of a clinical trial unwise,
- early evidence of benefit or harm of the experimental intervention

## Protocol amendments

Substantial amendments are only implemented after approval of the CEC and CA respectively.

Under emergency circumstances, deviations from the protocol to protect the rights, safety and well-being of human subjects may proceed without prior approval of the sponsor and the CEC/CA. Such deviations shall be documented and reported to the sponsor and the CEC/CA as soon as possible.

All Non-substantial amendments are communicated to the CA as soon as possible if applicable and to the CEC within the Annual Safety Report (ASR).

# Background and Rationale

## Background and Rationale

Heart rate variability (HRV) analysis is a possibility to examine the functioning of the autonomic nervous system (ANS). The ANS is divided into a sympathetic and parasympathetic branch which can stimulate or inhibit the heart trough the sinoatrial node. The HRV represents the length variance of the beat to beat intervals between the individual heart rates and allows to examine the power of the two branches. During the analysis of the HRV, the low frequency represents the sympathetic whereas the high frequency represents the parasympathetic part.

The sympathetic nerves leave the spinal cord at different levels whereas the parasympathetic nerves originate from the brain stem. Therefore a lesion of the spinal cord has an effect on the sympathetic part of the ANS. The higher the lesion the more the sympathetic part is affected.

The analysis of the HRV in spinal cord injured people shows partly different results than in able-bodied subjects. In a study the low frequency component is not shown in some high level injured people [20]. Other studies discovered the sympathetic part as well in high level injured people but with a reduced power [13, 20, 32].

Because of the fact that the sympathetic nervous system doesn’t leave the spinal cord over the whole length of the spine, in low level injured people the sympathetic nervous system is only affected to the 6^th^ thoracic vertebra. A lesion under this segment hasn’t a direct effect on the cardiac innervation of the ANS. Studies examining the HRV in low level injured people show the appearance of both components but with a reduced power compared to healthy reference persons.

Hence stimulants of the ANS have an effect on the two components of HRV. The impact of caffeine, which represents such a stimulant on the HRV, have been examined already in several studies with able-bodied people [18, 25, 29, 34]. There is a wide divergence in results and the effect of caffeine on HRV in able-bodied people is not yet resolved definitively [21]. Meanwhile, the impact of caffeine on the performance shows an improvement in the short time anaerobic zone.

The effect of a caffeine supplementation on HRV in spinal cord injured people has not yet been examined whereas the performance enhancing effect in this group of people could not yet be answered conclusively.

With our study we would like to generate more knowledge on the HRV measurements in spinal cord injured people and on the effect of a caffeine supplementation on the appearance of its components in this group of people compared to able-bodied persons.

Furthermore we want to verify the performance-enhancing effect of caffeine in able-bodied people and get more knowledge on the impact in spinal cord injured people.

With the new knowledge of our study we would be a step closer to the development of a possible new classification method for sports competition in spinal cord injured people. Furthermore we will get evidence on the influence of caffeine on HRV and the effectiveness in performing trough a caffeine supplementation.

In the future HRV could be a parameter for training design or for the diagnostics of over-training in athletes. So in combination with caffeine an effective training and competition schedule could possibly be developed.

## Investigational Product (treatment, device) and Indication

Caffeine is a substance found naturally in the leaves, beans and fruits of a variety of plants and is regularly consumed by ~90% of adults. The most common dietary source of caffeine is coffee but also coca cola and energy drinks contain a high amount of caffeine.

Until 2004, caffeine was on the list of banned substances with a critical value. But then caffeine was removed from this list because of further knowledge on performance enhancing effects of caffeine at specific doses. They realized that higher doses act as performance inhibiting substances.

Caffeine is absorbed within 30 to 45 min and it exerts a variety of different effects on our body. The major effects of caffeine are an enhanced muscle contraction, elevation of epinephrine and norepinephrine and so acting as a stimulant influencing bronchodilatation, higher alertness and suppressing fatigue.

A variety of different studies showed performance enhancing effects of caffeine on exercise performance of aerobic but also on short term, high-intensity anaerobic performance. Most of the people respond with a performance enhancing effect but others act as non-responders or show some negative effects. Thus effects of caffeine intake depend on the regularity of caffeine consume as habitual consumers benefit less from caffeine intake before exercise performance. New considerations for athletes indicate to abstain from caffeine one week before the competition to get the best possible benefit from caffeine consumption.

Caffeine is classified as an A supplement which means that scientific experience showed some performance enhancing effects in a healthy trained population group. No supplement on the A-list is considered as a performance enhancing product in all situations, sports or in all different population groups.

In very high doses (>6 mg/kg body mass) caffeine can provoke some adverse effects. Side effects are known as tachycardia, tremor, sleep disturbances, headache and irregular heart rate. As our doses will be lower than these high doses such side effects won`t be expected.

## Preclinical Evidence

Not relevant.

## Clinical Evidence to Date

Since quite a while, caffeine is known as an ergogenic supplement for sports performance of able-bodied athletes. It was shown in several studies that caffeine enhances performance in short-term high-intensity exercise performance. Due to altered physical conditions after a spinal cord injury (decreased gut motility, impaired ANS, etc.) studies conducted with able-bodied subjects cannot be transferred one-to-one to wheelchair athletes. Therefore, it is compulsive to investigate the effects of caffeine on exercise performance also in wheelchair athletes. In a recent study [17] we were not able to show such performance enhancing effects in paraplegic elite athletes in a 1500m wheelchair exercise test. That`s why we want to go further to investigate the effects of caffeine on the level of the ANS.

It is known [34] that HRV is changed after the intake of caffeine in able-bodied subjects but it is also known that HRV changes after a spinal cord injury [13]. It was shown [19] that not all components of the HRV were detectable in spinal cord injured subjects. Some of these components were missing in some subjects. Therefore, it may be possible, that caffeine won`t alter the HRV because of these missing components. So, one first point is to understand, if HRV also changes after the intake of caffeine in tetraplegic and paraplegic subjects and if there are any differences between the three population groups (able-bodied subjects, tetraplegic and paraplegic subjects). It was also shown [15] that HRV is quite good reproducible in spinal cord injured subjects.

From another tetraplegic caffeine study [31] we have the information, that epinephrine and norepinephrine concentration did not increase after the intake of caffeine in tetraplegic subjects even if there was an increase in paraplegic subjects. If there is no increase of these catecholamines, it is possible that in tetraplegic patients no change in HRV and no change in exercise performance could be detected. Hence, it would be very interesting to see the correlation between the increase of the catecholamines, the change of the HRV and the effect of caffeine on exercise performance between our three different population groups.

## Dose Rationale / Medical Device: Rationale for the intended purpose in study (pre-market MD)

We have chosen to supplement our subjects with a dose of 6 mg per kg body mass. This dose corresponds to the common used dosage in studies conducted with able-bodied subjects and it also corresponds to the international guidelines given for caffeine supplementation. These guidelines recommend a dosage of 3 to 6 mg/kg body mass of caffeine. As we want to conduct our spinal cord injury study with the common used dosage from able-bodied studies we did chose a 6 mg/kg body mass dosage.

Caffeine is administrated one hour before the exercise performance test as we expect the peak concentration of methylxantines (caffeine) in our bloodstream alter this time span. Also timing of supplementation is a main interest that’s why we have chosen the same timing strategy as in able-bodied studies. If epinephrine and norepinephrine concentrations are very low at this time point, it is considered to use a longer time interval after the supplementation in future studies with spinal cord injured population groups.

The amount of caffeine will be filled in gelatine capsules.

## Explanation for choice of comparator (or placebo)

As a placebo we use mannitol filled in gelatine capsules. The same number of gelatine placebo capsules will be administrated as for the caffeine capsules. As the total amount of mannitol (a sugar) is a very low dose carbohydrate amount, no additional effect of this placebo is expected.

Mannitol is a white crystalline solid and has the same colour as caffeine filled in our gelatine capsules. It is manly used to produce tablets of medicine.

## Risks / Benefits

**Caffeine administration**: The dosage of the caffeine supplementation corresponds to the dosage recommended in international and national guidelines. Therefore, the risk of caffeine administration is a very low risk. Nevertheless some adverse effects as headache, higher heart rate, tremor, nausea or tachycardia can possibly occur.

**Placebo administration:** There is no known risk for the ingestion of mannitol as a placebo supplement.

**Blood withdrawal:** Through the repeated blood withdrawal some local inflammations can occur. These little inflammations should pass away after a few days. If there is nothing special in the subject’s anamnesis no other risks should occur.

**3-min exercise performance test:** There is always a cardiac risk during high intensive exercise. As our subjects should be healthy and physically active, this risk is minimized. Our subjects undergo a health check in advance including screening of medical history. This additional intervention should further minimize the risk factors for cardiac problems.

**HRV:** No risk for the subject because these measurements are non-invasive.

**Wearing of a breathing mask:** There is no risk for wearing a breathing mask. It shouldn`t provide any problems for breathing during the HRV measurements and during the exercise testing.

**Diabetes:** To ensure that our subjects don`t suffer from any type of diabetes without knowing it, we will previously measure blood glucose concentration (see exclusion criteria).

**Benefits:** With the maximal exercise test (VO_2max_-test) the subjects get more knowledge about their general health and about their fitness. They will get to know their maximal heart rate and also the maximal performance output as well as their respiratory parameters during the testing. After this maximal exercise test they get the possibility for a training consultation where they get tips to enhance their physical fitness or their performance. After the caffeine/placebo testing phase they will know how their body reacts on the administration of caffeine in the epinephrine and norepinephrine system but also how heart rate variability changes through caffeine supplementation. The subjects will get these informations after the end of the study. They get to know if this caffeine dose acts as an ergogenic aid in their personal performance over a 3 min time. They get some personal advice for the usage of caffeine in their own sport. Additionally they get an insight in actual research with the spinal cord injured population.

## Justification of choice of study population

We will divide our 30 subjects into three different population groups. The first group is the group of 10 able-bodied subjects who will act as a control for our spinal cord injured subjects. These subjects have to be healthy and physically active but they are no elite athletes.

The second group of population will be the paraplegic subjects injured below Th8. Their spinal cord injury is sensory and motoric complete (AIS A). As the innervation of the heart and thus the regulation of heart rate is located around Th1 to Th6, we can be certain, that their heart is normally innervated. That means we should identify the same or almost the same outcome for HRV as for our able-bodied subjects. It will be very interesting to see the differences in the action of caffeine on the ANS but also on exercise performance compared to able-bodied subjects.

The third population group represents our tetraplegic subjects. They will be injured between C5 and C7 in a sensory and motoric complete way (AIS A). With the inclusion of this population we are sure, that these subjects are able to handle an arm crank ergometer and that their autonomic innervation of the heart is disturbed.

# STUDY OBJECTIVES

## Overall Objective

The overall objective of this study is to understand the influence of caffeine on HRV and on exercise performance of these different population groups especially the effects on the tetraplegic and paraplegic subjects. So, we want to know whether caffeine acts as an ergogenic aid over a 3 min all out test in spinal cord injured subjects and if HRV is altered through the ingestion of caffeine. It is possible that the low frequency component of the HRV does not alter in tetraplegic subjects due to the missing sympathetic innervation of the heart.

## Primary Objective

**Primary objective for HRV:** First of all we try to find out if caffeine supplementation alters the HRV at rest. From previous literature it is clearly stated that caffeine activates sympathetic nerves through the distribution of epinephrine and norepinephrine [31]. As in the HRV we can differentiate between components reflecting sympathetic and parasympathetic nerve activation, HRV should alter after the intake of caffeine supplementation. In our tetraplegic subjects there is a lack in the sympathetic innervation. Therefore, it is possible, that HRV or more precisely the sympathetic component of the HRV does not alter after the intake of caffeine. The following questions arise: What is the difference between paraplegic and able-bodied subjects in HRV after caffeine supplementation? Are there any differences comparing paraplegic, able-bodied and tetraplegic subjects with each other? Or does caffeine even not influence HRV in these different population groups?

**Primary objective for exercise performance:** Our aim is to investigate if caffeine enhances exercise performance in a 3 min exercise test at the arm crank ergometer. As studies with able-bodied subjects already showed some performance enhancing effects our focus lays on the spinal cord injured populations. Due to physiological adaptations after the spinal cord injury gut motility is slowed down and ANS activity is impaired. Therefore, absorption and also action of caffeine may be altered or even retarded. That`s why we want to know, if caffeine shows the same ergogenic effects on exercise performance in the spinal cord injured population as it shows in able-bodied subjects.

## Secondary Objectives

As secondary objectives we want to test if caffeine increases catecholamine concentrations in the bloodstream in all three population groups and if there are any differences between these three groups.

## Safety Objectives

The study aims to assess the tolerability of caffeine in spinal cord injured subjects in terms of gastrointestinal and other side effects.

# STUDY OUTCOMES

## Primary Outcome

**Heart rate variability:** HRV difference between the measurement before the ingestion of caffeine compared to the measurement one hour after the ingestion of caffeine. This primary outcome gives us some further information about the effect of caffeine on the HRV and shows the differences between our three population groups.

**Exercise performance test:** We want to compare the power output between the two tests (placebo vs. caffeine) to know whether caffeine shows some ergogenic effects in the spinal cord injured population groups concerning arm crank exercise performance. To date, only one study examined exercise performance tests with the spinal cord injured population groups using caffeine as a supplementation [17]. These findings would provide some further information about caffeine supplementation in wheelchair sports.

## Secondary Outcomes

**HRV:** As a secondary outcome we try to compare HRV baseline measurement with the measurement after the exercise test. As we know from previous data, HRV should be altered after an exercise performance and could be influenced by the ingestion of caffeine.

**Catecholamines:** We will compare epinephrine and norepinephrine concentrations before the ingestion of any supplement with the concentrations one hour after the ingestion of the supplements. As some previous studies showed no effect of caffeine on epinephrine and norepinephrine concentrations in tetraplegic patients, it will be very interesting to see these results and to compare it to our HRV and performance exercise testing.

## Other Outcomes of Interest

**Blood pressure:** We will compare the baseline measurement of diastolic and systolic blood pressure before the ingestion of any supplement with the measurement one hour after the ingestion of the supplement. Maybe spinal cord injured subjects could benefit from the ingestion of caffeine with a faster blood pressure adaptation if they have to transfer from the supine to the sitting position.

**Heart rate:** We will compare heart rate during the exercise test between the two different supplementation trials (placebo vs. caffeine). As caffeine acts as a stimulant heart rate may be altered after the ingestion of caffeine compared to placebo.

**Rate of perceived exertion (RPE):** We will compare the RPE after the exercise performance test between the two intervention trials (placebo vs. caffeine). As caffeine is well known as a pain decreasing agent it is possible that RPE is lowered after the ingestion of caffeine compared to placebo.

**Tidal volume:** We intend to investigate the difference between the tidal volume during the HRV measurement before the ingestion of any supplement with the measurement one hour after the ingestion of the supplement. As caffeine shows some bronchodilatation effects it is possible that at the same breathing frequency the tidal volume is increased after the ingestion of caffeine compared to placebo.

**Lactate concentration:** As another outcome of interest we measure lactate concentration after the warm up, to control if the subject didn`t warm up too hard and to check that their warm up didn`t differ between the placebo and the caffeine trial. After the exercise performance test we will measure lactate concentration for a second time. We might see differences in lactate concentration between the two trials if they reach a higher power output in one of the two trials or maybe they can suffer more because of a reduced pain perception after the caffeine ingestion and that could possibly result in a higher lactate concentration.

## Safety Outcomes

In general, heart rate is monitored during the testing phase. If any problems occur (very fast heart rate or very low heart rate) we would recognize it and could react immediately. In case of any emergency, a doctor in the sports medicine would be available at all times. Additionally, a defibrillator is available in a distance of 20m from the test location. The study staff is equipped with a telephone for emergency cases. The Swiss Paraplegic Centre has its own emergency number if medical aid is required. Also blood pressure is measured regularly. After every test session the participant will be asked if he suffered from gastrointestinal or other side effects and if he thinks that he got placebo or caffeine before the test.

# STUDY DESIGN

## General study design and justification of design

Our study will be conducted in a placebo-controlled, double-blind and randomised study protocol. In total every subject visits the Sport Medicine for four times whereas every visit takes 2 hours expect for the first one where only 30min are needed. Every subject has to undergo three test sessions at the Institute of Sports Medicine in Nottwil. Before these three sessions the subject will visit the Institute of Sports Medicine in Nottwil for recruitment issues which involves study informed consent and a health questionnaire with specific questions about diseases and family health history including a measurement of blood glucose level.

Then at the screening, the subjects have to perform a maximal exercise test (ramp protocol at the arm crank ergometer) to measure maximal oxygen uptake, maximal heart rate and maximal power output. After a little break, subjects have to perform a familiarization trial at the arm crank ergometer (Angio V2, Lode, Groningen, Netherlands). They have to perform exactly the same test as they have to perform later in the two test sessions.

Minimum two days and maximum two weeks later the subjects visit the Sports Medicine Nottwil for the first test session. At first, they have to answer some specific questions about sleep, recent training, recent nutritional intake and the actual health condition. Afterwards, they have to lie down for a 10 min rest where after 9 min blood pressure will be measured. HRV will be measured during 5 min the supine position and 6 min in the sitting position by means of a heart rate monitor. Paced breathing (15x/min) [12] is mandatory and will be paced through a metronome. During the HRV measurement also tidal volume will be measured. As the first HRV measurement session is finished, a venous blood withdrawal will be taken to measure catecholamine concentrations. Then, when all these measurements are completed, a supplement (either caffeine or the placebo) will be ingested in gelatine capsules. After the ingestion of these capsules, the subject has a 40 min break where they can read something or just relax. During these 40 min physical activity or the ingestion of any nutrition is prohibited.

After these 40 min, the second HRV measurement starts. The process of this measurement is exactly the same as for the first HRV measurement. They lay down for a 10 min rest with blood pressure measurement after 9 min. Afterwards again a 5 min HRV measurement in supine position and a 6 min measurement in sitting position will be performed. Tidal volume is measured during this HRV measurement.

When this second HRV measurement session is finished, a second blood withdrawal has to be taken. That means, one hour after the ingestion of either placebo or caffeine, catecholamines are analysed for a second time. After blood sampling, the warm up can start. After the standardised warm up lactate concentration of the blood is analysed taking 10 µl of blood from the earlobe using a blood lactate analyser (Biosen, EKF diagnostic, Cardiff, Great Britain) for analysis. The subject will be asked to evaluate the warm up by rating of the perceived exertion (following the Borg scale from 6 to 20). Then the 3 min exercise test can start. During this test, respiratory parameters will be measured using a device to measure oxygen consumption (MetaMax 3B,Cortex Biophysik GmbH, Leipzig, Germany). Just after having finished these 3 min of exercising, for a second time lactate concentration will be measured and again they have to rate the exertion using the Borg scale (6 to 20).

After a short 5 min break the third HRV measurement starts. Again HRV will be measured during a 5 min time in supine position and a 6 min time in sitting position. Tidal volume will be measured during the whole HRV measurement. With this HRV measurement the first test session is finished and a second test session will be conducted at least more than two days later in a randomised order.

The first visit will take 30 min. The screening and familiarization trial will be finished in around 75 min. For the two test sessions the subject has to be at the Sports Medicine for about 90 min.

In the following chart, the two 90 min test sessions are visually displayed.


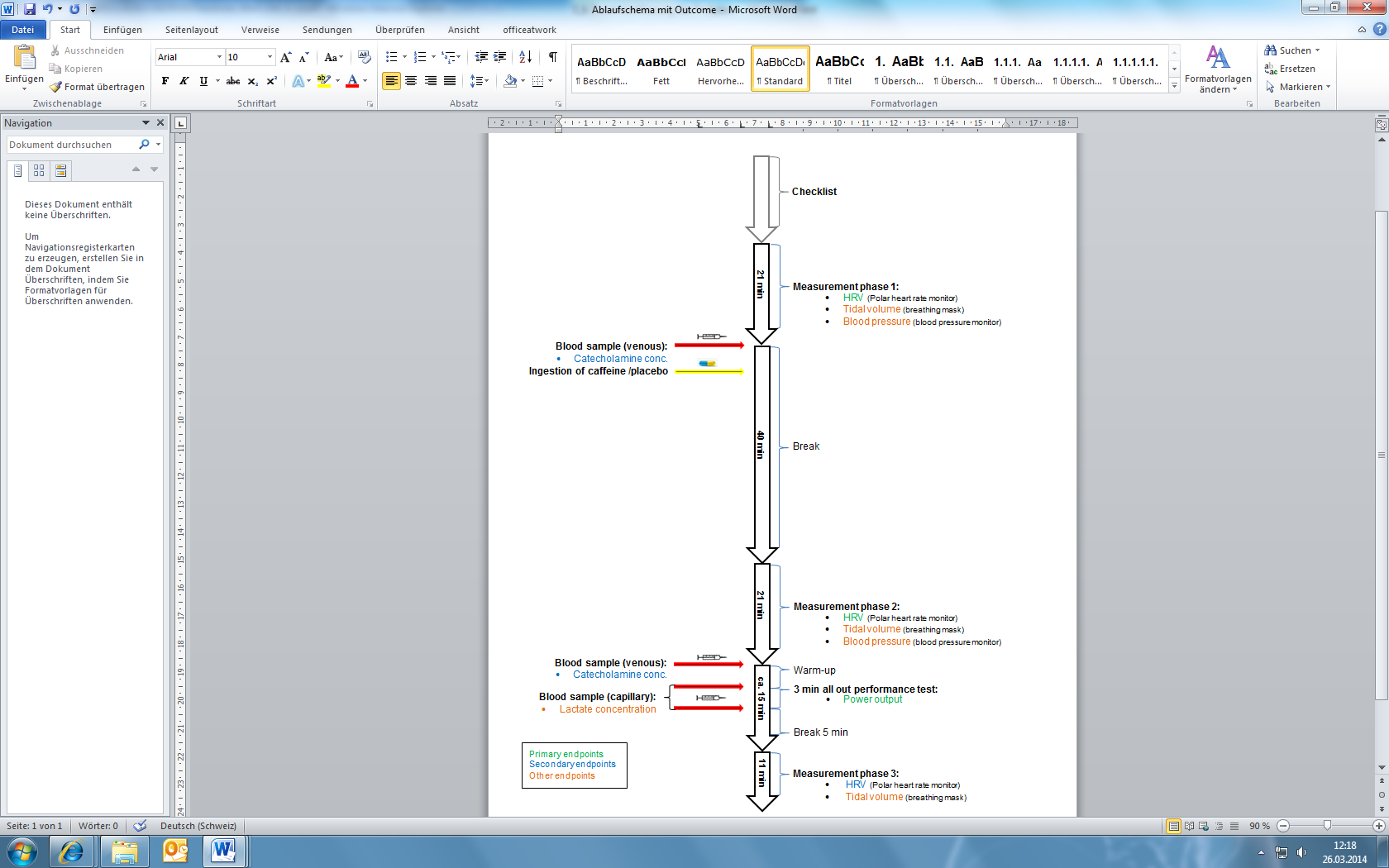


As we know from previous studies conducted with disabled subjects, the level of the lesion is one critical point. Therefore, we tried to include subjects with almost the same lesion level and sensory and motor complete lesion. This could help us to have more homogenous groups of different populations.

## Methods of minimising bias

### Randomisation

Randomisation will be assessed by lot. 50 % of all the subjects will first conduct the placebo trial and the other 50% first the caffeine trial. With which trial they start will be assigned by lot. So, there will be an “A” lot and a “B” lot and the fortune will decide whether they have the A or the B trial first. The test administrator only knows whether they have to ingest supplement A or supplement B but he does not know which supplement it is.

Applying a significance level of 0.05 and a power of 0.8 with a difference of 7.59 and a standard deviation of 10 Watt, we receive a total sample size of 9. With inclusion of a 10% drop-out rate we obtain a total sample size of 10 subjects per group. With the three different groups (able-bodied, paraplegic and tetraplegic subjects) we have in total 30 subjects and thus 120 visits overall.

### Blinding procedures

The pharmacist will define which capsules contain caffeine and which mannitol. He will name it A or B supplement. The test administrator then just see the A or B capsules and delivers the right capsule at the test day to the subject.

The real classification of the A or B supplement to caffeine or placebo will be written on a paper in an envelope by the pharmacist and will not be open before the end of the analysis of the data.

Therefore, the study will be double-blinded as neither the participant nor the test administrator and the principal investigator know which supplement the subject has ingested.

### Other methods of minimising bias

After the recruitment visit the subjects will visit the Institute of Sports Medicine in Nottwil performing the 3 min performance test for familiarization purposes.

## Unblinding Procedures (Code break)

The code for the blinding will be stored in a sealed envelope at the principal investigators office. Just the principal investigator or the physician are allowed to open this envelope in case of an adverse event. Otherwise this envelope will be opened after termination of the statistical analysis of the data.

# STUDY POPULATION

## Eligibility criteria

Participants fulfilling all of the following inclusion criteria are eligible for the study:

- Informed Consent as documented by signature (Appendix Informed Consent Form)
- Age between 18 and 60 years
- Healthy, non-smoking men
- Min. 3x45 min physical activity per week
- Spinal cord injured people:
  - AIS A (sensory and motoric complete injury)
  - termination of first rehabilitation
  - >6 months after the trauma
  - tetraplegic subjects: C5-7
  - paraplegic subjects: sub Th8

The presence of any one of the following exclusion criteria will lead to exclusion of the participant:

- Hypersensitivity to caffeine
- Caffeine 12 hours before the test session
- Women
- Other clinically significant concomitant disease states (e.g., diabetes, renal failure, hepatic dysfunction, cardiovascular disease, etc.),
- Known or suspected non-compliance, drug or alcohol abuse,
- Inability to follow the procedures of the study, e.g. due to language problems, psychological disorders, dementia, etc. of the participant,
- Participation in another study with investigational drug within the 30 days preceding and during the present study,
- Previous enrolment into the current study,
- Enrolment of the investigator, his/her family members, employees and other dependent persons,
- Less than 7 hours of sleep in the night before the testing sessions 1 and 2
- Not following the given nutrition guidelines

## Recruitment and screening

The participants will be recruited by the principal investigator and also by the other staff (Joëlle Flück and Fabienne Schaufelberger). Recruitment is intended to be orally or in written form by the recruitment sheet. Some subjects will get the recruitment sheet to email and other will directly be informed by their sports team (rugby, rowing, wheelchair athletics, hand cycling etc.). The selected participants have to undergo a health check with specific questions about their health status and risk factors and a blood sugar measurement. If the blood sugar test indicates any problems or health issues, the participant will be excluded from the study.

## Assignment to study groups

50 % of all the subjects will first conduct the placebo trial and the other 50% first the caffeine trial. With which trial they start will be assigned by lot. So, there will be an “A” lot and a “B” lot and the fortune will decide whether they have the A or the B trial first. The test administrator only knows whether they have to ingest supplement A or supplement B but he don`t know which supplement it is.

Each participant gets a number, his study ID. This ID will be written on every CRF to identify the participant. The participants name with his convenient ID will be stored in a sealed envelope to be sure that anonymity is given.

## Criteria for withdrawal / discontinuation of participants

Subjects have to be withdrawn if they won`t sign the written informed consent or if they won`t fulfil the study criteria. There is a definition of some criteria (sleep, nutritional intake 12h before the test, training intensity of previous training sessions, caffeine intake before the test, etc.). If these criteria won`t be fulfilled, then we have to withdraw the subjects from our trials.

Other reason for withdrawal would be if diseases occur or if the participant suffers from high gastrointestinal side effects or even some worse health problems because of the caffeine intake. If a participant is in danger, we have to withdraw him from our study for safety reasons.

# STUDY INTERVENTION

## Identity of Investigational Products (treatment / medical device)

### Experimental Intervention (treatment / medical device)

**Caffeine capsules**

The pharmacy of the Swiss Paraplegic Centre in Nottwil will provide us with gelatine capsules containing 50 mg or 100 mg of white caffeine powder. Capsules will be manufactured according to the GMP guidelines by the pharmacy. The dosage for each subjects will be calculated according to 6 mg per kg body mass. As we can only provide 50 mg or 100 mg capsules we have to round the actual dosage up or down which means an actual dosage between 5.7 and 6.3 mg/kg body mass.

This small difference in dosage is not relevant for our study and was successfully applied in several former studies.

### Control Intervention (standard/routine/comparator treatment / medical device)

**Mannitol capsules (Placebo)**

The pharmacy of the Swiss Paraplegic Centre in Nottwil will provide us with gelatine capsules containing either 50 mg or 100 mg of white mannitol powder for the placebo treatment. Capsules will be manufactured according to the GMP guidelines by the pharmacy and will be administered orally in the same amount as the caffeine capsules (see above).

### Packaging, Labelling and Supply (re-supply)

The capsules will be delivered by the pharmacy (see above) and are packed in a brown plastic vial provided by the pharmacy according to the common GMP guidelines. The vial will be labelled also according to the GMP guidelines. A vial contains a total of 60 capsules. If necessary, products will be re-supplied via pharmacy.

### Storage Conditions

The capsules are stored in two different boxes. One box with the “A” supplement and the other box with the “B” supplement (either caffeine or placebo). They are stored at room temperature and not exposed to light. There is only limited access to the storage area, where only study personal will have a key.

## Administration of experimental and control interventions

### Experimental Intervention

The caffeine capsules will be ingested one hour before the exercise test in a dose of 6 mg/kg body mass. The normal uptake time for caffeine is 30 to 45 min. As our spinal cord injured subjects suffer from a decreased gut motility a one hour timing period should be equivalent. About 15 min before the test the second HRV measurement will start. That will be 45 min after the ingestion of the caffeine capsule and should be in the range of the caffeine uptake. As a brewed coffee contains around 80 mg of caffeine for a 60 kg human (360 mg when supplementing 6 mg/kg BW) that means an intake of 4.5 coffees. For numerous subjects this is not excessive just a normal daily amount.

### Control Intervention

As a placebo we use gelatine capsules too. They have the same colour and the same size and are filled with mannitol (sugar alcohol). They cannot be distinguished from the caffeine capsules. The dose is the same as for the caffeine capsules. That means, that we hand the subjects the same amount of gelatine capsules for placebo as for caffeine. Even the timing is the same as for the caffeine. The capsules are ingested one hour before the exercise performance test.

## Dose / Device modifications

The dose won`t be changed. If there are any problems with the ingested dose, we will exclude the participant from the study.

As the caffeine dose is just administrated once we cannot change the dose during the study protocol.

## Compliance with study intervention

As our participants are in our laboratory, we will watch them ingesting the gelatine capsules. With this strategy we can be sure, that they have ingested our supplements at the specific given time point and that the whole dose was correctly ingested. All other tests will be conducted in our laboratory at the Institute of Sports Medicine in Nottwil and therefore every test is under our control.

## Data Collection and Follow-up for withdrawn participants

If a subject withdraws after session 1 or session 2 we will collect data but won`t use it in our analysis because of the missing data for session 3. Therefore, already collected data will be destroyed.

As we have no follow-up part for this study we won`t collect any data of withdrawn subjects.

## Trial specific preventive measures

No medication is permitted which influences heart rate or HRV. Subjects with diabetes (influences HRV) will be excluded from the study. As such patients often don`t know about their disease, we will conduct a measurement of the blood sugar to exclude diabetes mellitus in all our subject. Medicaments, which are often used by spinal cord injured people, such as antispasmodic (i.e. Lioresal) or bladder medication (i.e. Detrusitol) are permissible. Thereby it isn’t allowed to change the dosage of a medication during the study. Not permissible is the intake of any stimulants (i.e. Ephedrin). Dietary requirements are asked before every test session through a specific questionnaire. No intake of caffeine is allowed in the last 12 hours before the session. Nutrition should be kept standardised and is recorded before every test session too. As nutritional and caffeine intake could potentially influence our study outcome their recording and standardisation plays a central role.

## Concomitant Interventions (treatments)

Not applicable.

## Study Drug / Medical Device Accountability

The pharmacy of the Swiss Paraplegic Centre in Nottwil will produce the gelatine capsules filled with either caffeine or mannitol. They will put the expiry date on the packing. A pharmacist will then control the capsules and mark it as an “A” or “B” supplement for the blinding process. The pharmacy will produce enough capsules for the whole study. They will deliver us with 120 capsules of 100 mg and 60 capsules of 50 mg for each supplement (A or B). We calculated an average weight of 80 kg per subjects which means a dosage of 480 mg. Therefore we need for each subject 4x100 mg and 2x50 mg (30x4 100 mg=120 capsules; 30x2 50 mg=60 capsules).

Therefore we don`t have to worry a lack of capsules.

## Return or Destruction of Study Drug / Medical Device

In case of any redundant capsules, they will be returned to the pharmacy and will be destroyed.

# STUDY ASSESSMENTS

##
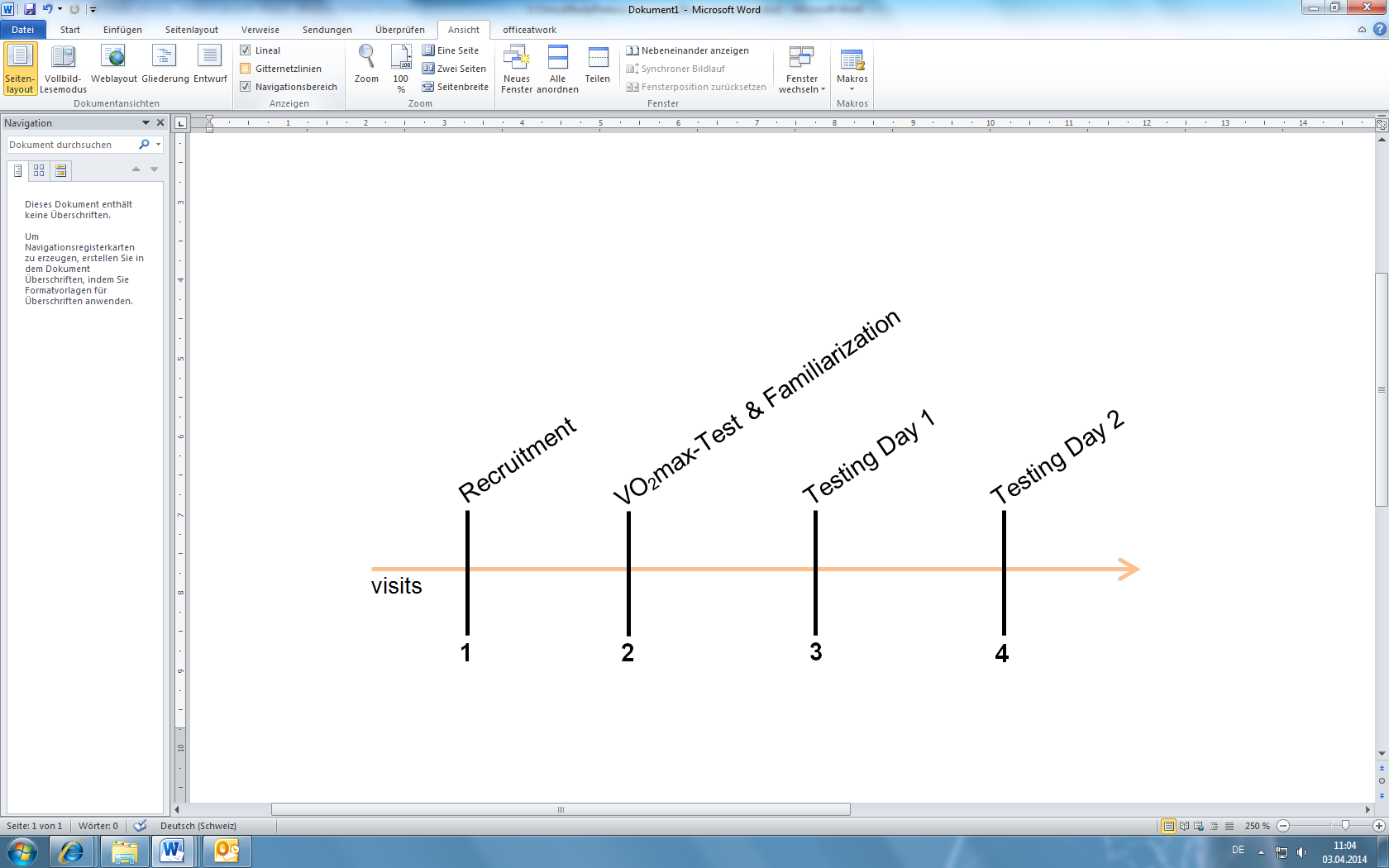
Study flow chart(s) / table of study procedures and assessments

## Assessments of outcomes

**HRV:** Measurement of the time interval between the separate heart rates using a Polar heart rate monitor (Polar, Kempele, Finland). Data is directly saved as R-R-Interval and will be transferred later on a computer for analysing the data. Heart rate variability will be analysed with the Kubios Software (University of Eastern Finland, Kuopio, Finland). Measurement will be done three times each in supine (for 5 min) and in sitting (for 6 min) position.

**Exercise performance test:** Assessment of power output. The subject will perform a 3 min all out performance test on an arm crank ergometer (Lode, Angio V2, Netherlands).

**Catecholamines:** Measurement of the concentration of epinephrine and norepinephrine in the blood. The blood samples are taken at the crook of the arm and they will be analysed later by Synlab in Lucerne for assessing the concentration of epinephrine and norepinephrine.

**Blood pressure:** Measurement of the systolic and diastolic arterial pressure using a blood pressure monitor (boso medistar S, Bosch + Sohn GmbH, Jungingen, Germany).

**Heart rate:** Heart beats per minute will be measured with the heart rate monitor (Polar, Kempele, Finland) and a heart rate belt (Polar, Kempele, Finland).

**Rate of perceived exertion (RPE):** This parameter is assessed by using the Borg scale from 6 to 20, whereas 6 represents no physical exertion and 20 represents a maximal exertion.

**Tidal volume:** This parameter measures the volume between inspiration and expiration. The volume is assessed by a breathing mask and a spirometer (MetaMax 3B,Cortex Biophysik GmbH, Leipzig, Germany).

**Lactate:** Measurement of the concentration of lactate in the capillary blood. The blood samples are taken at the ear lobe and will be directly analysed using blood lactate analyzer (Biosen, EKF diagnostic, Cardiff, Great Britain).

**HRV:** The values of HRV comparing before and after ingestion are measured 2 times at each of the testing day. This two measurements will be assessed with the following procedure: the subject is relaxing supine on a couch for 10 min. Then he starts breathing with a predefined breathing frequency of 15 breaths per minute. The assessment with fixed breathing rate takes 5 min in supine and 6 min in sitting position. The first baseline measurement takes place at the beginning of the experiment, the second after ingestion of caffeine or placebo followed by a 40 min break. For assessing the HRV we use a Polar heart rate monitor (Polar, Kempele, Finland) and a heart rate belt which transfers the data directly to the watch.

**Exercise performance:** We want to measure the power output during a 3 min all out performance test on an arm crank ergometer (Angio V2, Lode, Groningen, Netherlands). This test takes place after the ingestion of caffeine or placebo followed by a 40 min break and a short warming up.

### Assessment of secondary outcomes

**HRV:** For comparing the HRV baseline values with the values after the performance test, we use the results of the first HRV measurement (see above 9.2.1.) as baseline values. Then we perform a second measurement after the 3 min performance test followed by a 5 min break. For this measurement the subjects breaths with fixed rate in supine position on a couch for 5 min and then gets up with his upper body, sitting for 6 min with the same breathing rate. Meanwhile the HRV is measured by a Polar heart rate monitor (Polar, Kempele, Finland) and a heart rate belt. The data is transferred directly to and saved on the watch. The data will later be analysed on the computer with the Kubios Software (University of Eastern Finland, Kuopio, Finland).

**Catecholamine:** Epinephrine and norepinephrine are stress hormones and their concentration will be assessed twice during the testing procedure. The first one after the first HRV measurement as a baseline value and the second about 60 min after ingestion of the capsules. The subject is sitting up and the blood is taken at the crook of the arm. The blood samples are then frozen and will be later analysed by a laboratory.

### Assessment of other outcomes of interest

**Blood Pressure:** This parameter assesses the arterial blood pressure during the systolic and diastolic phase of the heart cycle. The blood pressure will be assessed twice. The first measurement takes place before ingestion of capsules in the 9^th^ minute of the relaxing phase of HRV measurement. The second measurement takes place again in the 9^th^ minute of the relaxing phase but of the second HRV measurement. A blood pressure monitor is used. The subject is in a supine position. The blood pressure is assessed only once at each measurement point.

**Heart rate:** This parameter assess the heart beats per minute and is measured during the HRV measurement and during the performance testing using the Polar heart rate monitor (Polar, Kempele, Finland). The heart rate during the HRV measurement are saved on the watch and will later be transferred on the computer. The values during the performance test will be directly transcribed on the CRF.

**Rate of perceived exertion (RPE):** The RPE scale is a method to measure subjectively the intensity of perceived exertion. The scale, called the Borg scale, runs from 6 to 20, whereas 6 represents no physical exertion and 20 represents a maximal exertion. The RPE will be assessed after warming up and after the 3 min performance test. The subject will be requested to rank verbally the intensity of warming up respectively of the performance test on the Borg scale.

**Tidal volume:** The tidal volume is used for measuring the breathing volume during normal inhalation and exhalation without any extra effort of the subject. This parameter is assessed during the three HRV measurements at the beginning of the testing procedure, after the 40 min break and after the 5 min break due to the performance testing. During the all out 3 min test, the respiratory parameters are recorded as well. The subject puts on a breathing mask which is connected to a spirometer (MetaMax 3B,Cortex Biophysik GmbH, Leipzig, Germany). The average volume during the time period of the HRV measurement is recorded.

**Lactate:** Measurement of lactate in the blood will be done twice during one experiment. The first time after the warm-up period on the arm crank ergometer (Angio V2, Lode, Groningen, Netherlands) and the second time after the 3 min performance test. The concentration of lactate will directly be measured by blood lactate analyzer (Biosen, EKF diagnostic, Cardiff, Great Britain).

### Assessment of safety outcomes

#### Adverse events

45 min after the capsules ingestion the subject will be asked for the tolerance to the caffeine/placebo ingestion and whether any gastrointestinal problems appeared.

#### Laboratory parameters

The blood sugar concentration will be assessed at the first recruitment meeting with the participants. After filling out the informed consent the blood glucose level will be measured using blood glucose meter (mylife Pura, Bionime, Berneck, Switzerland) at the Institute of Sports Medicine Nottwil. We use the postprandial blood sugar limit for determination of the threshold values. The subjects are included if the blood sugar level is ≤11.2 mmol/l. In case of higher values, the measurement is subsequently repeated for a second time to be sure for verification.

#### Vital signs

**Heart beat:** is measured during the first measurement phase and again apart from the second HRV measurement till the end of the experiment.

**Blood pressure:** is assessed twice every time at the 9^th^ minute of the relaxing period during the HRV measurement.

### Assessments in participants who prematurely stop the study

No follow-up period is intended due to the short half-life period of caffeine (max 5h).

## Procedures at each visit

**Recruitment:** The subject is visiting the Institute of Sport Medicine in Nottwil for the first time during the study period. Together with the subject the informed consent is filled in. After that the health questionnaire is answered and we check if all the inclusion and exclusion criteria are fulfilled including a blood glucose testing.

**Screening:** The subject performs a VO_2max_-Test on the arm crank ergometer for measuring the maximal rate of oxygen consumption. After that the subject has a 20-30 min break. Then the 3 min performance test of the following two testing day is carried out for familiarization purposes.

**Testing Day 1:** The subject arrives at 09:00 at the Institute of Sport Medicine in Nottwil. The check list is worked trough. After that the subject is requested to empty his bladder and in case of compression stockings to take them off. Then he puts on the heart rate belt and transfers himself in a supine position on a couch where a breathing mask is put on his mouth and his nose. The HRV and tidal volume measurements are performed. Then the breathing mask is taken off followed by taking of a blood sample at the crook of the arm and the ingestion of the capsules filled with caffeine or placebo. After that the subject has a 40 min break whereas it is not allowed to be physical active or to consume any beverage or food. According to this break, the breathing mask is put on again and the second HRV and tidal volume measurement is performed. After that a second venous blood sample is taken. Then the subject transfers himself to the arm crank ergometer where a short warming up is performed followed by taking a capillary blood sample on the ear lobe for analysing the amount of blood lactate. Then the 3 min all out performance test is performed. Afterwards the subject’s capillary blood is taken again and he has 5 min for transferring himself again on the coach and the HRV and tidal volume is measured for the last time. After this, the breathing mask and the heart rate belt are taken off.

**Testing Day 2:** The procedure of this second testing day is the same as on testing day 1 (see above) except to the kind of capsule which the subject has to ingest. So if the subject ingested the caffeine capsules on the first testing he has now to ingest the placebo capsules or the other way round.

### Split into subtitles by type of visit

Recruitment

• Filling in the informed consent

• Filling in the health questionnaire

• Blood glucose testing

Screening:

• VO_2max_-test

• Familiarization with the 3 min performance test

### Split into subtitles by type of visit

Testing Day 1 and 2:

• Filling in the checklist

• Subject empties his bladder and takes off the compression stockings

• HRV and tidal volume measurement on a couch (3 times)

• Sampling venous blood (twice) for catecholamine analysis

• Sampling capillary blood (twice) for lactate determination

• Ingestion of caffeine or placebo capsules

• 3 min all out performance test

# SAFETY

Due to the fact that we examine the effect of caffeine, which is defined as a supplement, there’s no appropriate section for our study. So we listed the applicable paragraph on the section drug studies.

## Drug studies

During the entire duration of the study, all serious adverse events (SAEs) are collected, fully investigated and documented in case report forms (CRF). Study duration encompassed the time from when the participant signs the informed consent until the last protocol-specific procedure has been completed.

### Definition and assessment of (serious) adverse events and other safety related events

A **Serious Adverse Event (SAE)** is classified as any untoward medical occurrence that:

- results in death,
- is life-threatening,
- requires in-patient hospitalization or prolongation of existing hospitalisation,
- results in persistent or significant disability/incapacity, or
- is a congenital anomaly/birth defect.

In addition, important medical events that may not be immediately life-threatening or result in death, or require hospitalisation, but may jeopardise the patient or may require intervention to prevent one of the other outcomes listed above should also usually be considered serious. [ICH E2A]

Examples of such events are intensive treatment in an emergency room or at home for allergic bronchospasm, blood dyscrasias or convulsions that do not result in hospitalisation, or development of drug dependency or drug abuse.

SAEs should be followed until resolution or stabilisation. Participants with ongoing SAEs at study termination (including safety visit) will be further followed up until recovery or until stabilisation of the disease after termination.

Assessment of Causality

Both Investigator and Sponsor-investigator make a causality assessment of the event to the study drug, based on the criteria listed in the ICH E2A guidelines:

| Relationship | Description |
| --- | --- |
| Definitely | Temporal relationship  Improvement after dechallenge*  Recurrence after rechallenge  (or other proof of drug cause) |
| Probably | Temporal relationship  Improvement after dechallenge  No other cause evident |
| Possibly | Temporal relationship  Other cause possible |
| Unlikely | Any assessable reaction that does not fulfil the above conditions |
| Not related | Causal relationship can be ruled out |
| *Improvement after dechallenge only taken into consideration, if applicable to reaction | |

Note that other categories can be used. However, a definition has to be provided in the protocol.

Unexpected Adverse Drug Reaction

An “unexpected” adverse drug reaction is an adverse reaction, the nature or severity of which is not consistent with the applicable product information (e.g. Investigator’s Brochure for drugs that are not yet approved and Product Information for approved drugs, respectively). [ICH E2A]

Suspected Unexpected Serious Adverse Reactions (SUSARs)

The Sponsor-Investigator evaluates any SAE that has been reported regarding seriousness, causality and expectedness. If the event is related to the investigational product and is both serious and unexpected, it is classified as a SUSAR.

Note: In case of double-blinded studies, unblinding is needed in order to determine a SUSAR.

Assessment of Severity

Describe the severity grading scale in use for this study, depending on the type of study and disease, the grades for severity described in the “Common Terminology Criteria for Adverse Events CTCAE Version x” terminology may be used and should be referred to here. Other definitions and grades are possible and shall be provided in the protocol (e.g. grading scale with explanation or reference to source).

### Reporting of serious adverse events (SAE) and other safety related events

The events will be reported on the CRF(side effects) by the person conducting the experiment during the experiment. Due to the short half-life period of caffeine no reporting after the testing is intended.

In case of any health related problems at the same day as the testing day, the subjects will be requested to inform the sponsor investigator Dr. Claudio Perret by phone.

Reporting of SAEs

All SAEs must be reported immediately and within a maximum of 24 hours to the Sponsor-Investigator of the study. The Sponsor-Investigator will re-evaluate the SAE and return the form to the site.

SAEs resulting in death are reported to the local Ethics Committee (via local Investigator) within 7 days.

Reporting of SUSARs

A SUSAR needs to be reported to the local Ethics Committee (local event via local Investigator) and to Swissmedic for category B and C studies (via Sponsor-Investigator) within 7 days, if the event is fatal, or within 15 days (all other events).

Reporting of Safety Signals

All suspected new risks and relevant new aspects of known adverse reactions that require safety-related measures, i.e. so called safety signals, must be reported to the Sponsor-Investigator within 24 hours. The Sponsor-Investigator must report the safety signals within 7 days to the local Ethics Committee (local event via local Investigator).

Periodic reporting of safety

No periodic reporting is intended.

### Follow up of (Serious) Adverse Events

No follow-up period is intended due to the short half-life period of caffeine (max 5 h).

# STATISTICAL METHODS

This study is a pilot-study with a small sample size. Therefore, normal distribution of the data cannot be assumed. Descriptive statistics, i.e. median, 25/75% quartiles, 95% confidence intervals and range, will be used. A p-value of < 0.05 will be considered significant, even though multiple hypotheses will be tested.

The treatment period (i.e. sequence of placebo and caffeine treatment) will not be included into the statistical analysis, because the wash-out period (48h) is considered more than long enough to prevent any carry-over effect. Furthermore, the period effect is considered to be negligible, because study participants will undergo a training session before the collection of data in order to avoid any learning effect.

## Hypothesis

In order to investigate the effect of caffeine on the performance and the HRV, the two following primary hypotheses will be tested:

1. H_0_: m_1_ = m_2_ versus
   H_1_: m_1_ ≠ m_2_
   where m_1_ = performance (power output) under placebo
    m_2_ = performance (power output) under caffeine

   This hypothesis will be tested for the three subject groups (i.e. tetraplegic, paraplegic, able-bodied).
2. H_0_: m_1_ = m_2_ versus
   H_1_: m_1_ ≠ m_2_
   where m_1_ = change in HRV from baseline to post-treatment under placebo treatment
    m_2_ = change in HRV from baseline to post-treatment under caffeine treatment

   This hypothesis will be tested for the three subject groups (i.e. tetraplegic, paraplegic, able-bodied).

## Determination of Sample Size

This study represents a pilot study and therefore no power analysis was performed.

A total of 36 subjects who are classified into 3 groups (tetraplegic, paraplegic, able-bodied) will be included which includes an over-recruitment by approximately 20% to take into account possible drop-outs. A minimum of 10 data sets will be required for each subject group.

## Statistical criteria of termination of trial

Not applicable

## Planned Analyses

### Datasets to be analysed, analysis populations

Only data from subjects who have completed the whole study protocol will be used for analysis. For each group, a minimum of 10 data sets will be analysed.

Analyses will be performed separately for the three subject groups (i.e. tetraplegic, paraplegic, able-bodied). No direct comparison between the groups will be performed.

### Primary Analysis

For testing the first hypothesis

H_0_: m_1_ = m_2_ versus
H_1_: m_1_ ≠ m_2_
where m_1_ = performance (power output) under placebo
 m_2_ = performance (power output) under caffeine

the Wilcoxon signed-rank test will be used to compare the performance under placebo with that under caffeine for three subject groups separately.

For testing the second hypothesis

H_0_: m_1_ = m_2_ versus
H_1_: m_1_ ≠ m_2_where m_1_ = change in HRV from baseline to post-treatment under placebo treatment
 m_2_ = change in HRV from baseline to post-treatment under caffeine treatment

the Wilcoxon signed-rank test will be used to compare the performance under placebo with that under caffeine for three subject groups separately.

### Secondary Analyses

The correlation between HRV and performance under caffeine will be evaluated with the Spearman's rank correlation coefficient.

Changes in tidal volume, blood pressure and epinephrine and norepinephrine blood concentration from baseline to post-treatment values will be compared between placebo and caffeine treatment using the Wilcoxon signed-rank test.

Changes in lactate blood concentration under treatment before and during performance testing will be compared between placebo and caffeine treatment using the Wilcoxon signed-rank test.

Changes in tidal volume and HRV under treatment before and after performance testing will be compared between placebo and caffeine treatment using the Wilcoxon signed-rank test.

### Interim analyses

No interim analyses will be performed.

### Safety analysis

Not applicable.

### Deviation(s) from the original statistical plan

Not applicable.

## Handling of missing data and drop-outs

All available data will be analysed and no missing data will be replaced. Only data from full sets of data will be analysed and drop out will be replaced by new study participants.

# QUALITY ASSURANCE AND CONTROL

## Data handling and record keeping / archiving

### Case Report Forms

All data are recorded with a paper CRF except the HRV values, heart rate during HRV measurement and the catecholamine concentration. These values will be directly transferred in secuTrial after analysing on a computer respectively in a laboratory. For each enrolled study participant a CRF is maintained and the CRF is kept current to reflect subject status at each phase during the course of the study. Subjects cannot be identified in the CRF by name or initials and birth date. The following coded identification is used: for example HRV_T_01, which means the subjects will be identified by his belonging to the group (paraplegic, tetraplegic or able-bodied group) and a number which identifies him within the group.

Every staff member is authorized for any CRF entry and by initials noted on the CRF the investigator can be subsequently identified. The data on the CRF will later be entered in the secuTrial database (iAS, Berlin, Germany). So a double data entry will occur.

One person will enter the data from the CRF paper form into the secuTrial database. A second person will control the correctness of the data in the secuTrial and the principal investigator will finally release the data for archival storage.

The Clinical Trial Unit will only give access to three people: Joëlle Flück and Fabienne Schaufelberger will get access for data entering and controlling and the principal investigator Claudio Perret for data release.

### Specification of source documents

Source data in this study consider: the health questionnaire, the Informed Consent Form, the randomisation number, the checklists before testing.

Data that are directly recorded in the CRF or the excel sheet are: blood pressure, tidal volume, RPE, heart rate during 3 min performance test, lactate concentration, power output.

### Record keeping / archiving

All study data must be archived for a minimum of 10 years after study termination in a specific lockable room at the Institute of Sports Medicine Nottwil and electronic in secuTrial.

## Data management

The secuTrial (iAS, Berlin, Germany) web-based data capture and management system will be used. The system is hosted on a secure hospital server. Personal data, which allows to identify an individual, will not be stored on a server or personal computer. These data will be printed on paper and stored in a locked data storage room with limited access. An anonymous identification number will allow to connect the data entered in the system with an individual. Only anonymous data will be stored in the electronic data capture and management system.

The data management system allows to define roles for data entry, verification, validation and management. An audit trail documents all entries and changes made in the system and by whom.

Data will first be collected using paper CRF, except for heart rate variability which will be printed out on an anonymous paper sheet. All data is then transferred into the data management system secuTrial. Plausibility rules will be established to promote data quality. Furthermore, data will be reviewed and verified prior to data entry completion.

### Data Management System

The secuTrial (iAS, Berlin, Germany) web-based data capture and management system will be used. The system is hosted on a server of the Swiss Paraplegic Centre and only accessible through the Swiss Paraplegic Group’s intranet.

The project set-up and eCRFs will be tested by the Data Manager of the Swiss Paraplegic Clinical Trial Unit prior to the release into the productive environment using a custom testing protocol.

### Data security, access and back-up

During the course of the study, the individuals mentioned in this application will have access to the data according to their pre-defined roles.

After termination of the study and archiving of the data, only the Principal Investigator and secuTrial Data Manager will have access to the data. The Data Manager will only have access to the anonymized data.

A back-up system is set in place. It is maintained and controlled by the IT department of the Swiss Paraplegic Centre.

### Analysis and archiving

Anonymized data will be exported from the data management system for analysis in statistic software.

Electronic data will be stored in an electronic archive maintained and controlled by the IT department of the Swiss Paraplegic Centre. Electronic data will not be deleted. Source data and identification data will be stored in a locked archive room with limited access for a minimum of 10 years.

### Electronic and central data validation

Plausibility rules will be established in the data capture system to promote data quality. Furthermore, data will be reviewed and verified prior to data entry completion.

## Monitoring

The Clinical Trial Unit of the Swiss Paraplegic Centre will independently perform regular monitoring visits prior to the start and during the study. The CTU will have access to every testing procedure or data sampling procedure. All questions will be answered by the study staff.

## Audits and Inspections

All study documentation and the data are accessible to auditor or inspectors and questions will be answered during regular inspections.

## Confidentiality, Data Protection

Direct access to source documents are only permitted for purposes of monitoring, audits and inspections. The study staff will have access at any time of the study in dependence on the authorization by secuTrial.

## Storage of biological material and related health data

The blood samples will be analysed at Synlab Lucerne and will be destroyed after analysis.

# PUBLICATION AND DISSEMINATION POLICY

The study is intended to be published in a peer-reviewed journal. The authorship will be registered in the following order: Flück J., Schaufelberger F., Schäfer D., Wilhelm M., Strupler M., Krebs J., Perret C.

# FUNDING AND SUPPORT

## Funding

The Institute of Sports Medicine in Nottwil will bear majorly the expenses for the study.

## Other Support

Not applicable.

# INSURANCE

This study is a category A study with low risk for the participants. So liability insurance will be provided by the Sponsor. A copy of the certificate is filed in each investigator site file and the trial master file

# REFERENCES

1. Declaration of Helsinki, Version October 2013, (http://www.wma.net/en/30publications/10policies/b3/index.html )
2. International Conference on Harmonization (ICH, 1996) E6 Guideline for Good Clinical Practice. (http://www.ich.org/fileadmin/Public_Web_Site/ICH_Products/Guidelines/Efficacy/E6_R1/Step4/E6_R1__Guideline.pdf )
3. International Conference on Harmonization (ICH, 1997) E8 Guideline: General Considerations for Clinical Trials <http://www.ich.org/fileadmin/Public_Web_Site/ICH_Products/Guidelines/Efficacy/E8/Step4/E8_Guideline.pdf>)
4. Humanforschungsgesetz, HFG Bundesgesetz über die Forschung am Menschen (Bundesgesetz über die Forschung am Menschen, HFG) vom 30. September 2011/ Loi fédérale relative à la recherche sur l’être humain (loi relative à la recherche sur l’être humain, LRH) du 30 septembre 2011. (<http://www.bag.admin.ch/themen/medizin/00701/00702/07558/index.html?lang=de>)
5. Verordnung über klinische Versuche in der Humanforschung (Verordnung über klinische Versuche, KlinV) vom 20. September 2013 / Ordonnance sur les essais cliniques dans le cadre de la recherche sur l’être humain (Ordonnance sur les essais cliniques, OClin) du 20 septembre 2013. (http://www.bag.admin.ch/themen/medizin/00701/00702/12310/index.html?lang=de)
6. Heilmittelgesetz, HMG Bundesgesetz über Arzneimittel und Medizinprodukte (Heilmittelgesetz, HMG) vom 15. Dezember 2000/Loi fédérale sur les médicaments et les dispositifs médicaux (Loi sur les produits thérapeutiques, LPT) du 15 décembre 2000. (<http://www.admin.ch/ch/d/sr/8/812.21.de.pdf>)
7. ISO 14155:2011 Clinical investigation of medical devices for human subjects -- Good clinical practice (www.iso.org)
8. ISO 10993 Biological evaluation of medical devices (www.iso.org)
9. WHO, International Clinical Trials Registry Platform (ICTRP) (http://www.who.int/ictrp/en/)
10. Acharya UR, Joseph KP, Kannathal N, Lim CM, Suri JS. Heart rate variability: a review. Medical and Biological Engineering and Computing 44(12): 1031-1051. 2006
11. Agiovlasitis S, Heffernan KS, Jae SY, Ranadive SM, Lee M, Mojtahedi MC, Fernahall B. Effects of paraplegia on cardiac autonomic regulation during static exercise. American Journal of Physical Medicine & Rehabilitation 89(10): 817-823. 2010
12. Aubert AE, Seps B, Beckers F. Heart rate variability in athletes. Sports Medicine 33(12): 889-919. 2003
13. Bunten DC, Warner AL, Brunnemann SR, Segal JL. Heart rate variability is altered following spinal cord injury. Clinical Autonomic Research 8(6): 329-334. 1998
14. Castiglioni P, Rienzo MD, Veicsteinas A, Parati G, Merati G. Mechanisms of blood pressure and heart rate variability: an insight from low-level paraplegia. American Journal of Physiology 292(4): R1502-1509. 2006
15. Ditor DS, Kamath MV, MacDonald MJ, Bugaresti J, McCartney N, Hicks AL. Reproducibility of heart rate variability and blood pressure variability in individuals with spinal cord injury. Clinical Autonomic Research 15(6): 387-393. 2005
16. Figoni SF. Cardiovascular and haemodynamic responses to tilting and to standing in tetraplegic patients: a review. Paraplegia 22: 99-109. 1984
17. Flueck JL, Mettler S, Perret C: Influence of Caffeine and Sodium Citrate Ingestion on 1500 m Exercise Performance in Elite Wheelchair Athletes: A Pilot Study. International Journal of Sports Nutrition and Exercise Metabolism : 2013 Nov 25. [Epub ahead of print]
18. Hibino G, Moritano T, Kawada T, Fushiki T. Caffeine enhances modulation of parasympathetic nerve activity in humans: quantification using power spectral analysis. The Journal of Nutrition 127(7): 1422-1427. 1997
19. Inoue K, Ogata H, Hayano J, Miyake S, Kamada T, Kuno M, Kumashiro M. Assessment of autonomic function in traumatic quadriplegic and paraplegic patients by spectral analysis of heart rate variability. Journal of the Autonomic Nervous System 54(3): 225-234. 1995
20. Kazuhiro I, Hajime O, Junichiro H, Shinji M,, Toyohiko K, Masahiko K, Masaharu K. Assessment of autonomic function in traumatic quadriplegic and paraplegic patients by spectral analysis of heart rate variability. Journal of the Autonomic Nervous System 54(3): 225-234. 1995
21. Koenig J, Jarczok MN, Kuhn W, Morsch K, Schäfer A, Hillecke TK, Thayer JF. Impact of caffeine on heart rate variability: a systematic review. Journal of Caffeine Research 3(1):22-37. 2013
22. Koh J, Brown TE, Beightol LA, Ha CY, Eckberg LD. Human autonomic rhythms: vagal cardiac mechanisms in tetraplegic subjects. Journal of Physiology 474(3): 483-495. 1994
23. [13] Mohr T, van Soeren M, Graham TE, Kjaer M. Caffeine ingestion and metabolic responses of tetraplegic humans during electrical cycling. Journal of Applied Physiology 85(3): 979-985. 1998
24. Monad M, Viggiano A. Vicidomini C, Viggiano A Iannaccone T, Tafuri D, De Luca B. Expresso coffee increases parasympathetic activity in young, healthy people. Nutritional Neuroscience 12(1): 43-48. 2009
25. Nishijima Y, Ikeda T, Takamatsu M, Kiso Y, Shibata H, Fushiki T, Moritani T. Influence of caffeine ingestion on autonomic nervous activity during endurance exercise in humans. European Journal of Applied Physiology 87(6): 475-480. 2002
26. Notarius CF, Floras JS. Caffeine enhances heart rate variability in middle-aged healthy, but not heart failure subjects. Journal of Caffeine Research 2(2): 77-81. 2012
27. Otsuka Y, Shima N, Moritani T, Okuda K, Yabe K. Orthostatic influence on heart rate and blood pressure variability in trained persons with tetraplegia. European Journal of Applied Physiology 104(1). 75-78. 2008
28. Rimaud D, Calmels P, Pichot P, Bethoux F, Roche F. Effects of compression stockings on sympathetic activity and heart rate variability in individuals with spinal cord injury. The Journal of Spinal Cord Medicine 35(2): 81-88. 2012
29. Sondermeijer HP, van Marle AGJ, Kamen P, Krum H. Acute effects of caffeine on heart rate variability. The American Journal of Cardiology 90(8): 906-907. 2002
30. Takahashi M, Matsukawa K, Nakamoto T, Tsuchimochi H, Sakaguchi A, Kawaguchi K, Onari K. Control of heart rate variability by cardiac parasympathetic nerve activity during voluntary static exercise in humans with tetraplegia. Journal of Applied Physiology 103(5): 1667-1677. 2007
31. Van Soeren M, Mohr T, Kjaer M, Graham TE: Acute effects of caffeine ingestion at rest in humans with impaired epinephrine responses. Journal of Applied Physiology 80(3): 999-1005. 1996
32. Wang YH, Huang TS, Lin JL, Hwang JJ, Chan HL, Lai JS, Tseng YZ. Decreased autonomic nervous system activity as assessed by heart rate variability in patients with chronic tetraplegia. Archives of Physical Medicine and Rehabilitation 81(9): 1181-1184. 2000
33. Xhyheri B, Manfrini O, Mazzolini M, Pizzi C, Bugiardini R. Heart rate variability today. Progress in Cardiovascular Diseases 55(3): 321-33. 2012
34. Yeragani VK, Krishnan S, Engels JE, Gretebeck R. Effects of caffeine on linear and nonlinear measures of heart rate variability before and after training. Depression and Anxiety 21(3):130-134. 2005
35. Zamunér AR, Silva E, Teodori RM, Catai AM, Moreno MA. Autonomic modulation of heart rate in paraplegic wheelchair basketball players: linear and nonlinear analysis. Journal of Sports Science 31(4): 396-404. 2013

# APPENDICES

1. Caffeine Guidelines
2. Case Report Form
3. Checklist
4. Health Questionnaire
